# Discovery of Peptidic Ligands against the SARS-CoV-2 Spike Protein and Their Use in the Development of a Highly Sensitive Personal Use Colorimetric COVID-19 Biosensor

**DOI:** 10.1021/acssensors.2c02386

**Published:** 2023-05-30

**Authors:** Xingjian Yu, Bofeng Pan, Cunyi Zhao, Diedra Shorty, Lucas N. Solano, Gang Sun, Ruiwu Liu, Kit S. Lam

**Affiliations:** † Department of Biochemistry & Molecular Medicine, University of California, Sacramento, Sacramento, California 95817, United States; ‡ Department of Biological and Agricultural Engineering, University of California, Davis, Davis, California 95616, United States; § Department of Chemistry, University of California, Sacramento, Sacramento, California 95616, United States

**Keywords:** OBOC library, high-throughput screening, SARS-CoV-2 detection, nanofibrous membrane, at-home testing kits

## Abstract

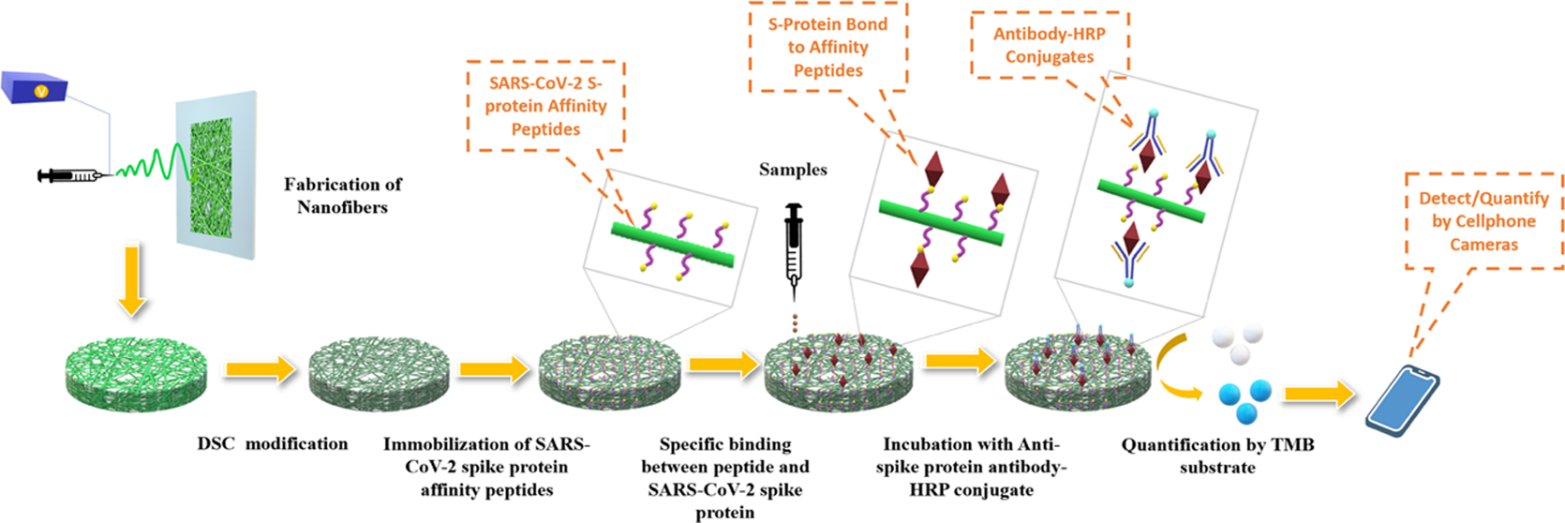

In addition to efficacious vaccines and antiviral therapeutics, reliable and flexible in-home personal use diagnostics for the detection of viral antigens are needed for effective control of the COVID-19 pandemic. Despite the approval of several PCR-based and affinity-based in-home COVID-19 testing kits, many of them suffer from problems such as a high false-negative rate, long waiting time, and short storage period. Using the enabling one-bead-one-compound (OBOC) combinatorial technology, several peptidic ligands with a nanomolar binding affinity toward the SARS-CoV-2 spike protein (S-protein) were successfully discovered. Taking advantage of the high surface area of porous nanofibers, immobilization of these ligands on nanofibrous membranes allows the development of personal use sensors that can achieve low nanomolar sensitivity in the detection of the S-protein in saliva. This simple biosensor employing naked-eye reading exhibits detection sensitivity comparable to some of the current FDA-approved home detection kits. Furthermore, the ligand used in the biosensor was found to detect the S-protein derived from both the original strain and the Delta variant. The workflow reported here may enable us to rapidly respond to the development of home-based biosensors against future viral outbreaks.

By April 2023, there have been more than 760 million cases of COVID-19 infection globally since its outbreak at the end of 2019. The accurate and timely diagnosis of COVID-19 is essential to map and monitor COVID-19 cases for necessary containment measures to combat the pandemic.^1,2^ Compared to diagnosis offered in clinical labs, rapid COVID-19 tests, such as point-of-care (POC) or self-administered household COVID-19 tests, can screen COVID-19 carriers from a vast population at the community level, which would significantly expedite the COVID-19 diagnosis and greatly reduce the workload of clinical labs. In addition, rapid tests can provide valuable information when clinical lab tests are not available or cannot be administered to patients.^3−5^

Many rapid testing kits have received regulatory approval from public health authorities worldwide for COVID-19 diagnosis.^6^ Based on the detection mechanism and target analytes, these rapid COVID-19 tests can be classified as the PCR assay and immunoassay. The real-time reverse transcription polymerase chain reaction (rRT-PCR) amplified trace RNA from the SARS-CoV-2 virus at the molecular level by billions of times, making it the gold standard for COVID-19 diagnosis.^7,8^ However, currently, many household rRT-PCR COVID-19 tests require sample shipment to certified clinical labs as the implementation of rRT-PCR outside the lab environment is confined by space and equipment. Besides, cross-contamination during the shipment could undermine the overall accuracy of testing, and the time-to-results can vary from several hours to days.^6^ COVID-19 immunoassays utilize monoclonal immunoglobulins to directly detect the presence of characteristic viral antigens from nasal swabs and saliva.^9^ The immunoassay is more convenient and flexible than rRT-PCR in terms of the instrument setup, which allows anywhere rapid COVID-19 tests with shorter time-to-results and no need for specialized equipment.^10^ However, the results from the immunoassay may not be as reliable as rRT-PCR tests because the accuracy and sensitivity of the immunoassay could not match with those of rRT-PCR due to variations of antigen levels in different samples and lower sensitivity.^11,12^ Nevertheless, the advantages and drawbacks of immunoassay make it complementary to rRT-PCR tests. The immunoassay is more valuable and practical as the preliminary approach to screening and rRT-PCR as the confirmatory test for SARS-CoV-2 viral infection.

We would like to develop a more sensitive and cost-effective viral antigen assay for in-home rapid high-throughput COVID-19 screening. One of the core elements of such viral antigen assay is the affinity elements that serve as the “super binders” to enrich characteristic motifs from the SARS-CoV-2 virus for detection. While most commercially available immunoassays aim at the nucleocapsid protein (N-protein), which is encapsulated inside the virus, transmembrane spike proteins (S-protein), which interact with angiotensin-converting enzyme 2 (ACE2) to initiate infection,^13^ might be a better target. Compared to the N-protein, the S-protein from the SARS-CoV-2 virus is more divergent than its counterpart in SARS-CoV-1 and MERS viruses. Besides, embedded on the viral surface as a homotrimer, the S-protein is probably more accessible for the affinity-based assay.^14,15^ So far, S-protein-targeting affinity elements used in SARS-CoV-2 detection include antibodies and their fragments,^16,17^ DNA aptamer,^18,19^ nanobodies,^20^ peptides,^21−23^ and ACE2 receptor analogue mini-proteins.^24,25^ Among these affinity elements, peptides demonstrate good chemical stability, processability, and scalability, making them promising candidates as the affinity elements for the development of sensitive and user-friendly household COVID-19 immunoassays. In addition, the platform in which these affinity elements are deployed can boost the assay’s performance and miniaturize the assay, affording more user-friendly biosensors. Microporous nanofibrous membranes have found unique advantages in the development of highly sensitive colorimetric sensors due to the structural characteristic of extremely high specific surface areas allowing increased loading of affinity elements, leading to dramatically intensified colorimetric signals high enough for human eye detection.^26−28^ The integration of both SARS-CoV-2 S-protein selective binding peptides and nanofibrous membranes could lead to the development of biosensors against COVID-19 with desired selectivity, sensitivity, and easy-to-use functions.

Here, we report a novel nanofibrous membrane-based biosensor utilizing S-protein binding peptides, discovered by the one-bead-one-compound (OBOC) combinatorial technology as the affinity element. Rooted in versatile solid-phase peptide synthesis on the microscale beads, the enabling OBOC technology created by Lam et al.^29^ involves (1) the generation of millions of peptides or other synthetic molecules through the split-and-mix synthetic strategy, such that each bead displays many copies of one unique compound, (2) rapid screening of the huge compound-bead library (millions diversity) against specific biological target(s), and (3) physical isolation of the positive beads for chemical identification of the library compound via direct Edman sequencing, mass spectrometry analysis, or chemical decoding.^30−32^ The OBOC platform is well suited for the rapid discovery of binding ligands against proteins derived from virions that cause global viral pandemics, such as COVID-19.^33^ We have recently discovered several peptidic ligands against the S-protein as the lead compounds from a 6-amino acid long random diverse OBOC linear peptide library via enzyme-linked colorimetric selection with an optimized protocol (Figure 1a). Based on the structural motifs of these leads, we designed OBOC-focused libraries for further optimization under higher stringency screening conditions, yielding ligands with nM binding affinity. These peptides can be covalently immobilized on the porous nanofibrous membrane to capture S-proteins that can be visualized by immunochromogen conjugates for naked-eye detection (Figure 1b). The limit of detection (LOD) of this peptide-coated nanofibril sensor can be as low as 2–10 ng/mL for the S-protein in a saliva sample, and the biosensor would allow rapid and cost-effective in-home COVID-19 self-diagnosis without additional equipment, labor, and sample processing, thus enabling this inexpensive COVID-19 diagnostic to be able to reach a broader range of the population.

**Figure 1:**
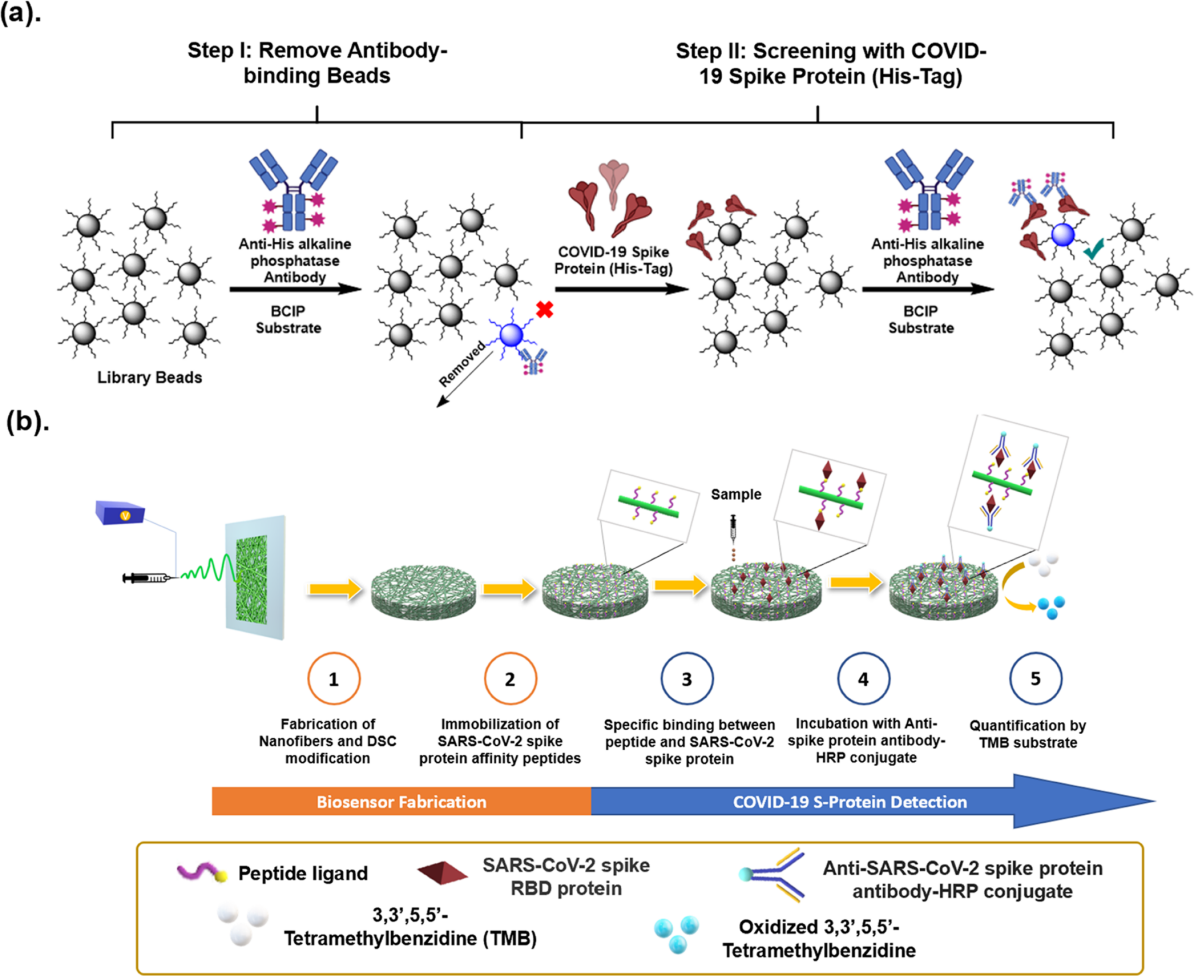
(a) Two-step OBOC combinatorial peptide library screening for SARS-CoV-2 virus spike protein affinity peptides using enzyme-linked colorimetric staining to visualize positive beads. (b) Preparation of biosensor for SARS-CoV-2 viral spike protein by grafting the affinity peptide onto the PVA-co-PE porous nanofibrous substrate and detection with the enzyme-linked colorimetric reaction.

## Experimental Section

### Materials

*N*,*N*′-disuccinimidyl carbonate (DSC), triethylamine (TEA), 1,4-dioxane, acetone, phosphate-buffered saline (PBS), 96-well plates, 5-bromo-4-chloro-3′-indolylphosphate *p*-toluidine (BCIP) powder, and anti-His-tag monoclonal antibody (3D5) alkaline phosphatase conjugate (His-AP) were purchased from Thermofisher Scientific (Pittsburgh, PA). Poly(vinyl-*co*-ethylene) (PVA-*co*-PE, PE content of 27%, MW_n_ = 90 000), bovine serum albumin (BSA), and trifluoracetic acid (TFA) were purchased from Sigma-Aldrich (Milwaukee, WI). All Fmoc-protected amino acids were purchased from P3 Biosystems (Louisville, KY) and Chem-Impex International, Inc. (Wood Dale, IL). TentaGel S NH_2_ resin (0.29 mmol/g NH_2_ loading) was purchased from Rapp Polymere (Tubingen, Germany). Rink amide MBHA resin (0.68 mmol/g NH_2_ loading) was purchased from P3 Biosystems (Louisville, KY). All chemicals and resins were used as received without purification. The wild-type SARS-CoV-2 spike protein receptor-binding domain (Catalog No. 40592-V08B, C-His-tag) and Delta-variant SARS-CoV-2 spike protein receptor-binding domain (L452R, T478K) (Catalog No. 40592-V08H90) were purchased from Sino Biological (Wayne, PA). The SARS-CoV-2 spike protein active trimer (Catalog No. SPN-C52H) was purchased from ACROBiosystems (Newark, DE). The spike proteins were reconstituted based on recommended conditions and aliquoted into different portions for storage at −80 °C.

### Synthesis of OBOC Libraries

The OBOC peptide library was synthesized using a split-and-mix strategy on the TentaGel resin by standard 9-fluorenylme-thyloxycarbonyl (Fmoc) chemistry. For one typical cycle of library synthesis, the TentaGel resin was divided into several portions by their volume. The number of portions equals the number of types of amino acids added in that cycle, and each portion of the resin received specific Fmoc-protected amino acid, 6 Cl-HOBt, and DIC at equal molarity, which is 5 times molar excess to primary amine on the TentaGel resin from that portion. Each coupling took 2 h at room temperature, and the completion of coupling was monitored by a ninhydrin test, which yielded a clear yellow supernatant for completed coupling. Different portions were then consolidated and washed with 3× DMF, 3× MeOH, and 3× DMF before adding 20% 4-methyl piperidine in DMF to remove the Fmoc protecting group for 5 min. The deprotection was repeated for another 15 min before the resin was washed by alternating DMF and MeOH for the next cycle. Once all cycles were finished, the resin was washed by DCM and completely dried by a pump. The global deprotection was performed by a TFA cocktail (TFA/H_2_O/triisopropylsilane = 95:2.5:2.5%, v/v) for 3 h. Then the TFA was drained, and the resin was washed with DMF, DCM, H_2_O, and EtOH throughout. The prepared library was stored in 70% EtOH in H_2_O for screening.

### Screening of the High-Affinity Peptide Binder Toward the SARS-CoV-2 S-Protein

For focus OBOC libraries L8, L9, and L10, 300 mg (∼1 million beads) of each library bead were washed with water and then three times with PBS, and blocking buffer (5% BSA, 0.5% Tween 20 in PBS, w/w) was then added and the libraries were incubated at 4 °C overnight. After that, the library beads were incubated with 5 mL of anti-His-AP (1–2 mg/mL, diluted 1:3000 with blocking buffer) for an hour. The buffer was then drained, and the library was washed with washing buffer (PBS, 0.5% Tween 20) three times, and 5 mL of the BCIP substrate dissolved in buffer C (0.165 mg/mL TBS, pH = 8.8) was added to stain the false-positive beads that interact with the AP conjugate alone. At this stage, beads that developed a dark blue color were physically removed under an optical microscope with a hand-held micropipette. The remaining library beads were washed with water and PBS buffer three times, followed by the addition of 5 mL of the COVID-19 S-protein receptor-binding domain (RBD) solution (5 nM). After 30 min of incubation, the S-protein solution was drained, and the library beads were washed with a glycine solution (1 mM, pH = 3.0) to remove the bound S-protein due to weak/nonspecific interactions. The color development was performed under the same conditions as the previous colorization step to visualize and confirm peptides that can bind to spike proteins as positive hits. These positive beads were picked and sequenced by an automatic Edman degradation microsequencer.

### Synthesis of Peptidic Affinity Elements and Affinity Characterization by the Biolayer Interferometry (BLI) Assay

Unless specified otherwise, all peptides were synthesized on the Rink amide resin by a microwave peptide synthesizer (CEM Liberty Blue 2.0) using Oxyma/DIC as coupling reagents. The cleavage of peptides was performed by a TFA cocktail (TFA/H_2_O/triisopropylsilane = 95:2.5:2.5%, v/v) for 2 h at room temperature. All peptides were purified by HPLC (Shimadzu LC-20AR) with a C18 reverse-phase column and characterized by a MALDI-TOF mass spectrometer (Bruker).

The biolayer interferometry assay (BLI) was performed by the Octet Bio-Layer Interferometry detection system (RED 384). 220 μL of the kinetic buffer blank, 220 μL of 1 μM biotinylated peptide solutions, and 220 μL of the S-protein solution at various concentrations prepared by serial dilution were added to 96-well plates. Streptavidin biosensors were first equilibrated in the kinetic buffer for 30 min before being loaded with biotinylated peptides as “bait” for 200 s. Then, these biosensors were dipped into kinetic buffer wells for baseline correction for 1 min, and dipped into spike protein wells with various concentrations to collect association kinetics data. After that, the biosensors were dipped into blank kinetic buffer wells to collect dissociation kinetics. The data was processed by ForteBio Data Analysis 8.1 software. Background signals were subtracted from reference wells where no spike protein was added, and then the beginning of the dissociation signal was aligned with the end of the association. Global fitting was conducted using the data from association and dissociation steps by the 1:1 model, which defines the dissociation constant (*K*_D_) as follows:
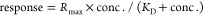


### Fabrication of PVA-*co*-PE Nanofibrous Membranes

The electrospinning method was used to prepare PVA-co-PE fibrous membranes with a nanoscale structure.^34,35^ PVA-*co*-PE polymer grains were dissolved in a mixture of water and isopropanol (weight ratio of 30:70%) at 85 °C with gently stirring for 6 h to obtain a transparent and homogeneous PVA-*co*-PE polymer solution with a concentration of 7 wt %. The polymer solution was then filled in four 20-mL syringes with metallic needles. The pumping rate was controlled by a syringe pump (Kent Scientific) at a speed of 2 mL/h. A high voltage of 28 kV (EQ 30, Matsusada Inc.) was applied to the metallic needles as the electrostatic source. The formed electrospun PVA-*co*-PE membranes were collected on a metallic roller covered with wax paper. The tip-to-collector distance was controlled at 15 cm.

### Modification of the PVA-*co*-PE Nanofiber with S-Protein Affinity Peptides

After the fabrication through electrospinning, 0.1 g of PVA-*co*-PE nanofibrous membranes were prepunched into 1 cm^2^ round pieces and immersed into a DSC modification solution (prepared by dissolving 2.5 g of DSC and 0.2 g of TEA in 50 mL of 1,4-dioxane). The mixture was stirred for 2 h at 70 °C. The modified membranes were thoroughly washed with 1,4-dioxane for 15 min twice and with acetone for 10 min and vacuum dried.

### SARS-CoV-2 Spike Protein Detection Assay

Paper-based sandwich ELISA was used in the study. 50 μL of the 0.5 mM biotinylated peptide (L10-2) with high affinity to the SARS-CoV-2 viral spike protein, as we demonstrated above, was added to the membrane platform and incubated for 20 minutes. Then, the membrane was exposed to 5% BSA to block the remaining nonspecific binding sites. Subsequently, in PBS or saliva, 50 μL of solutions containing varied concentrations (ranging between 0 ng/mL and 10 mg/mL) of the SARS-CoV-2 spike protein were added to the membranes and incubated for 30 min under gentle agitation, respectively. Then, 50 μL of the 250 ng/mL HRP conjugated antispike antibody was added to each membrane. After 15 minutes, the membranes were washed several times with PBS buffer and dried in air. 15 μL of the tetramethylbenzidine (TMB) substrate for the HRP substrate (ThermoFisher) was added to the membranes, and the membranes were settled in an LED lightbox (E mart). The colorimetric signal was captured by a smartphone (iPhone X) and analyzed using Photoshop (Adobe) software. The smartphone was held over nanofibrous membranes at a fixed distance of 50 cm to capture images of each result. The red channel (*R*-value) could be read through Photoshop’s color histogram. The *R*-values were correlated to the concentrations of the spike protein. The sample size of all experiments was 3.

The saliva samples were collected from three different lab members into 2 mL vials. Half of the pieces were centrifuged under 4000 rpm for 5 minutes, after which the supernatants were collected. Various concentrations of the spike protein (0–1000 ng/mL) were then mixed with saliva supernatants and directly added to the modified nanofibrous membranes following the protocol mentioned in the next section.

### Colorimetric Data Processing

After the colorimetric development with the TMB substrates was completed in 15 minutes, the membranes were placed in an LED lightbox (E mart), and images were captured through a smartphone camera. The *R* channel values of the region of interest (circles with a diameter of 40 pixels) were obtained using Photoshop (Adobe) software. The background of all images is a standard white Whatman paper with an *R-*value of 230 under the light intensity of 6500 lux. The ratio of colorimetric intensity was estimated as *B*/*B*_0_ = (*R*_max_ – *R*_x_)/(*R*_max_ – *R*_0_), where *R*_max_ is the *R*-value of the negative control group (no HRP), *R*_0_ is the *R*-value of the positive control group (without blocking), and *R*_x_ is the R-value at a specific concentration of the spike protein.

## Results and Discussion

### Design, Screening, and Optimization of the OBOC Library

**Figure 2:**
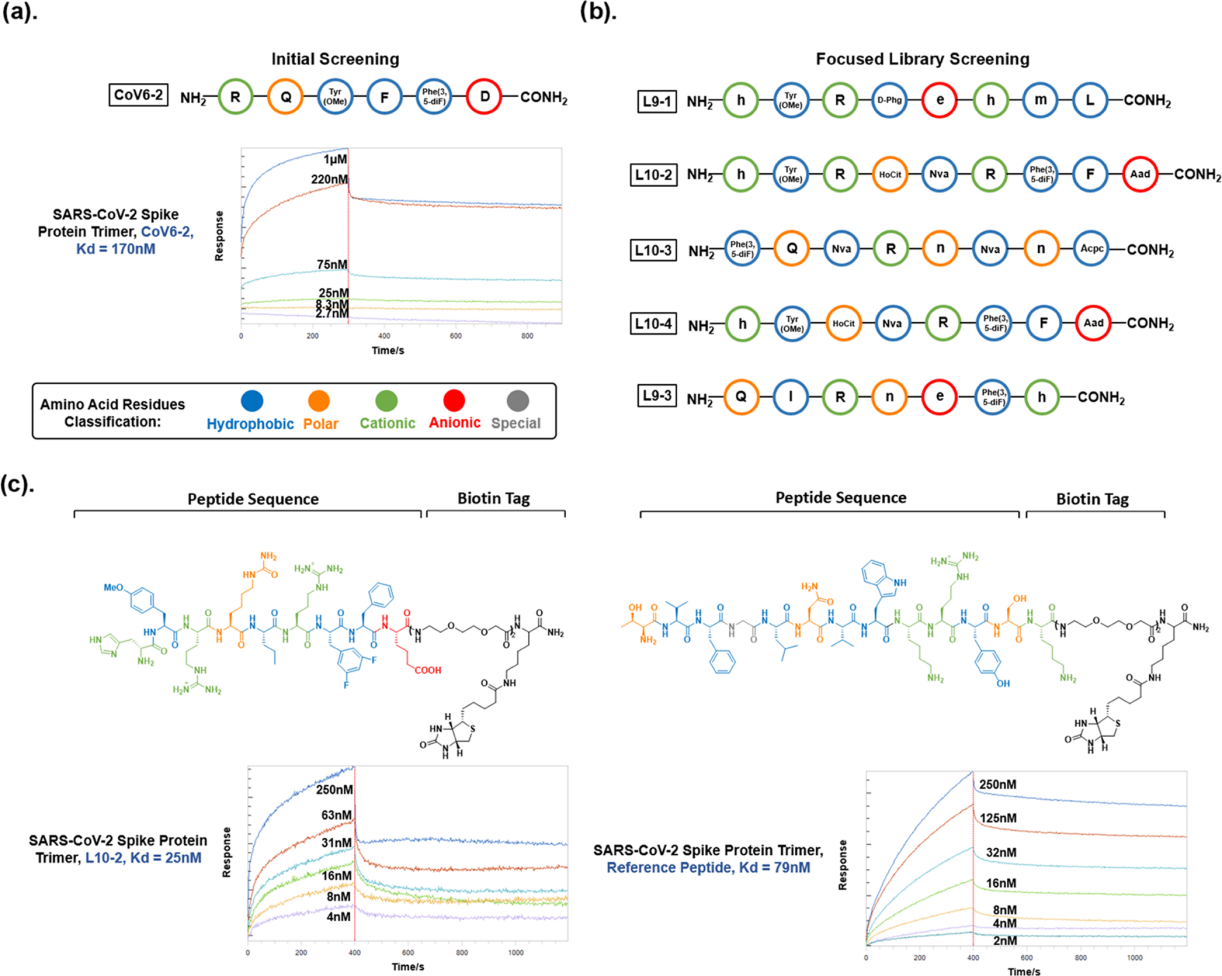
Binding affinities of SARS-CoV-2 binding peptides. (a) CoV6-2, the peptide with the highest affinity (170 nM) identified from the initial screening of diverse random OBOC peptide libraries. (b) Positive hits obtained from focused libraries screened under more stringent conditions. (c) Chemical structure of biotinylated peptides L10-2 and reference peptides reported by Pentelute et al. and the BLI assay that characterizes the binding kinetics.

Even with limited structural information on SARS-CoV-2 viral spike proteins available at the beginning of the pandemic, we started the OBOC screening campaign with a random linear hexapeptides library comprised of 32 canonical and noncanonical amino acids at each position (Figure S1 and Table S1) to discover peptidic ligands against the S-protein. We used the similar enzyme-linked immunocolorimetric method described in previous work^36^ to screen the OBOC library (Figure 1a). In short, we incubated the libraries with anti-His-AP conjugates first to identify peptides that bind to the antibody by BCIP substrates and upon addition, turned the conjugate-bound beads into a dark blue color. These beads could constitute the false-positive leads during the following screening with S-proteins so that they were removed with a 20 μL hand-held micropipette with an ultrathin micropipette tip. Subsequently, the libraries were incubated with His-tag S-proteins, and the positive beads with S-protein bound were visualized by adding another fresh batch of antibodies and substrates. We screened about 300,000 library beads and successfully discovered and sequenced five hexapeptide beads as our initial hits (Figure 2a and Table S2).

We then resynthesized these 6-mer peptides on the TentaGel resin to confirm their interactions with the S-protein. Under the screening condition, the confirmation study indicated that CoV6-2 was the strongest binding ligand. Typically, it is unlikely to discover peptides with nanomolar or even higher affinity in the first round of screening using a random diverse library.^37^ Therefore, in lieu of detail characterizing all peptide hits, it is a common practice to design focused libraries based on the promising leads from the initial round of screening.^38,39^ We selected CoV6-2 as the lead peptide for further optimization with the focused library approach. Nine focused libraries with different lengths were prepared (Figures S2–S4 and Tables S3 and S4), and d-amino acids were used abreast of l-amino acids to diversify enantiomeric isomerism as well as better resistance against proteolysis. The focused libraries were screened under more stringent conditions with much lower S-protein concentration (1 nM) and shorter incubation time (30 min) to ensure that only peptides with strong interaction were discovered and sequenced. During the optimization, we successfully discovered and sequenced six peptides against the spike protein from focused libraries (Figure 2a and Table S5). These peptides are shorter than previously reported S-protein RBD binding peptides (13-mer)^19^ and potentially have better solubility and stability against enzymatic degradation.

### Characterization of the Binding Affinity of Peptides Against the SARS-CoV-2 S-Protein

We synthesized the biotinylated peptides by inserting a Lys(biotin) unit and two 8-amino-3,6-dioxaoctanoic acid (AEEA) spacers at the C-terminus of the peptides for biolayered interferometry (BLI) analysis to determine the binding kinetics of these peptides with the SARS-CoV-2 spike protein (Figures 2b and S5). After sequence optimization, peptides L8-1, L9-1, L10-2, L10-3, and L10-4 were found to bind reasonably well with the S-protein active trimer. In all, peptide L10-2 exhibited the highest binding affinity (25 nM) toward the spike protein active trimer, which is ∼3 times stronger than a 13-mer COVID-19 affinity peptide (79 nM) reported by Pentelute et al.,^21^ which was synthesized and characterized as reference peptide. Additionally, the binding affinity of L10-2 was found to be 8 times higher than CoV6-2, the lead peptide we selected from the initial screening. Both CoV6-2 and L10-2 are able to bind to the Delta variant of spike protein RBD with similar affinity (Figure S6). Unlike antibodies, peptides are cheaper to produce and are quite stable to temperature and therefore have no cold-chain issue for home and field diagnostics. Compared to other *in silico* discovery methods,^22,23^ the OBOC technology can easily incorporate unnatural building blocks, such as d-amino acids and noncanonical amino acids, via chemical synthesis into the library design, which can be difficult for current *de novo* peptide design tools, which use primarily canonical amino acids.

### Fabrication and Modification of the Nanofibrous Membrane

**Figure 3:**
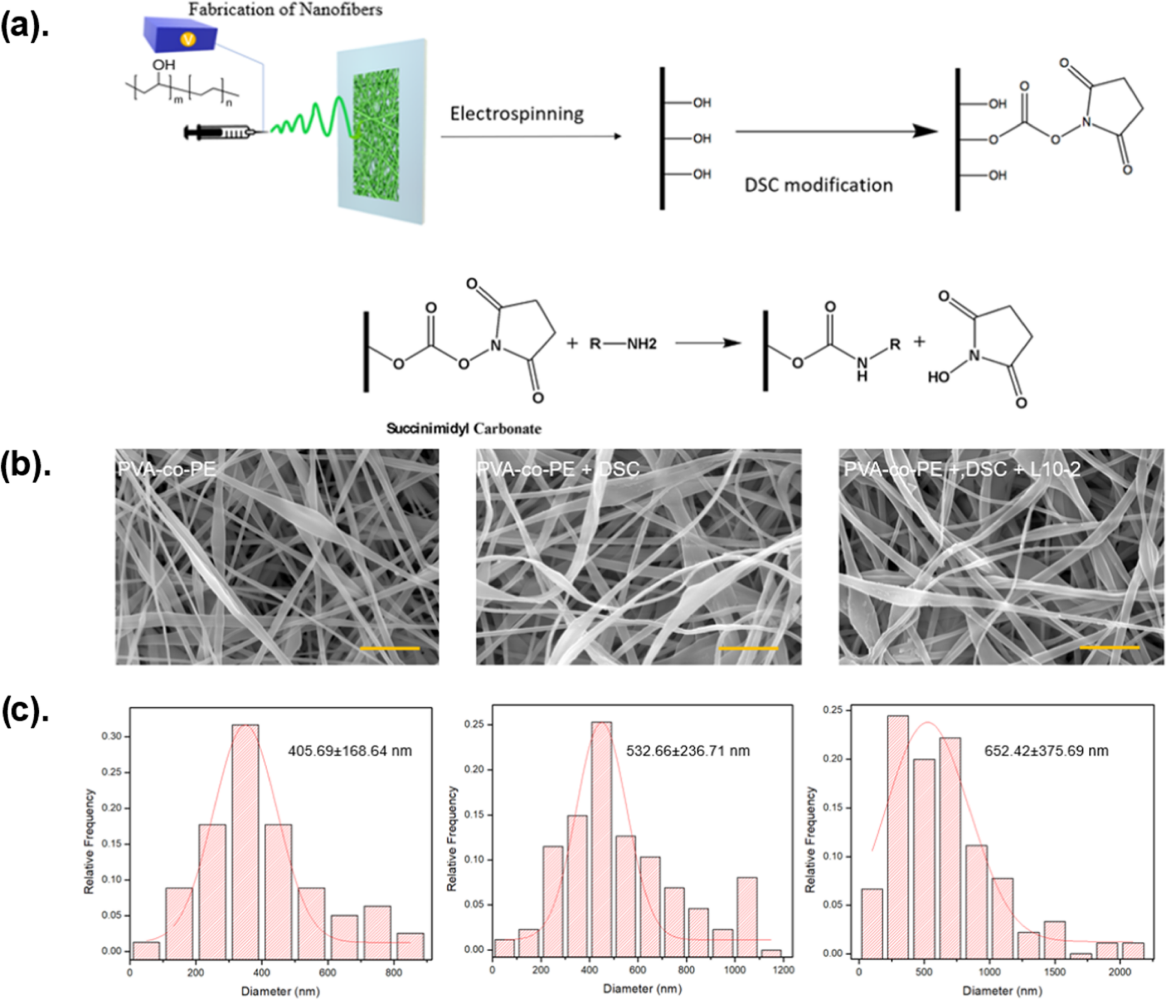
(a) Reaction scheme for peptide immobilization on the PVA-co-PE nanofibrous membrane (NFM). (b) SEM images of pristine PVA-*co*-PE NFM, NFM after *N*,*N*′-disuccinimidyl carbonate (DSC) modification, and NFM after the immobilization of the L10-2 peptide (scale bar: 5 μm). (c) Fiber diameter distribution of pristine PVA-*co*-PE NFM, NFM after DSC modification, and NFM after the immobilization of the L10-2 peptide.

We chose PVA-co-PE as the substrate material for fabricating COVID-19 biosensors because the hydrophilic vinyl alcohol and hydrophobic ethylene segments from the PVA-*co*-PE copolymer offer good mechanical strength, thermal stability, abundant reactive sites (−OH), and needed biocompatibility.^40^ In addition to these advantages, previous studies also indicated that the hydrophilic PVA-*co*-PE matrix exhibited minimized nonspecific protein adsorption, which has been a significant concern for the solid support materials used to capture biomarkers.^41^ The PVA-*co*-PE nanofibrous membrane was fabricated via electrospinning, producing nanosized fibers with an ultrahigh specific membrane area.^42^ The structure of nanofibrous membranes is characterized by scanning electron microscopy (SEM), with an average fiber diameter of ∼405.69 nm and microsize pores (Figures 3b and S7). The abundant hydroxyl group on nanofibrous membranes makes covalent linkage with biomolecules possible. Based on previous studies, the PVA-*co*-PE nanofibrous membrane chemically modified by *N*,*N*′-disuccinimidyl carbonate (DSC) showed a very high capacity for biomolecules with free amino groups (Figure 3a), and the chemically modified nanofibrous membranes kept their microporosity and nanofibrous structures intact before and after immobilization of peptides (Figure 3c), indicating their structural stability and allowing them to be used in subsequent steps of capturing targeting molecules.^43^

### Immobilization of Peptides Onto the Nanofibrous Membrane

Since peptide L10-2 demonstrated the highest binding affinity and better immobilization efficiency, we chose L10-2 as the candidate for the detection experiments. First, we qualitatively investigated the immobilization of L10-2 peptides on DSC-modified nanofibrous membranes. Biotinylated L10-2 (L10-2BT) was covalently immobilized on the DSC-activated nanofibrous membrane through nucleophilic substitution at the unique N-terminal amino group (Supporting Information S5). We then used streptavidin-Alexa647 conjugates to characterize the immobilization and distribution of L10-2BT on the nanofibrous membrane. Fourier-transform infrared spectroscopy (FTIR) revealed the successful immobilization of L10-2BT on the nanofibrous membrane. Fluorescence images taken by the Bio-Rad imaging system showed a strong contrast from the unmodified membrane and that the distribution of immobilized peptides was homogenous. In addition, the modified membrane showed a higher content of the immobilized L10-2 peptide than the 96-well plates (Figure S9).

### Performance of the Colorimetric Biosensor

After fabrication and surface modification by peptide L10-2, the resulting nanofibrous membrane was employed to perform naked-eye detection of the SARS-CoV-2 S-protein using the TMB/HRP-based colorimetric reaction as the reporter. We first ran different control assays to evaluate the specificity of biosensors (Figure S10). These experiments demonstrated minimal nonspecific interaction of the nanofibrous membrane toward various proteins (S-protein, antibodies, etc.). In addition, the membrane modified by the scrambled L10-2 sequence (SL10-2) was found to generate a much-reduced color response by the biosensor. We also saw a much weaker colorimetric response from non-nanofibrous films, suggesting that the PVA-co-PE nanofiber material has a much higher capacity for the grafting of affinity ligands and is needed for this rapid assay (Figure S11).

**Figure 4:**
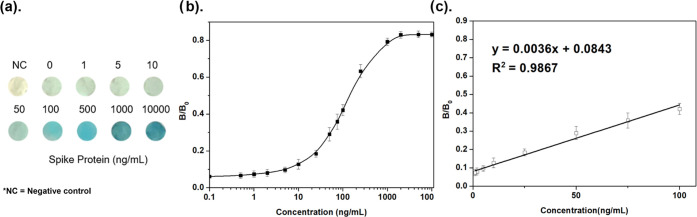
Sensitivity of the colorimetric spike protein assay. (a) Optical images of nanofibrous membranes in the detection of the SARS-CoV-2 S-protein over a range of protein levels. (b) Corresponding *B*/*B*_0_ value and SARS-CoV-2 S-protein concentration. By examining color intensity via Photoshop software following the equation of *B*/*B*_0_ = (*R*_max_ – *R*_x_)/(*R*_max_ – *R*_0_), where *R*_max_ is the *R*-value of the negative control group (no HRP), *R*_0_ is the *R*-value of the positive control group (without blocking), and *R*_x_ is *R*-value at a specific concentration of the S-protein. (c) Linear equation for the colorimetric assay was fitted to be *y* = 0.0036*x* + 0.0843 (*R*^2^ = 0.9867) between 1 and 100 ng/mL. The lowest distinguishable (via Photoshop) concentration of the S-protein was 1 ppb, and the limit of detection (LOD) was calculated to be around 18.3 ng/mL using the equation of LOD = 3.3σ/*S*, where σ is the standard deviation of the response and *S* is the slope of the calibration curve.

To explore the sensitivity of the biosensor, we ran the detection assay at various SARS-CoV-2 S-protein concentrations (0–10 000 ng/mL), and the naked-eye readout for the blue color at different concentrations is shown in Figure 4a. The faint light blue color from the negative control (0 ng/mL) suggests a possible nonspecific weak interaction between the peptides and antibody-HRP conjugates.^44^ For naked-eye detection, the discernible color change was found to start at ∼10 ng/mL. The blue color intensified as the level of the S-protein increased until 1000 ng/mL, beyond which the color change became indistinguishable, possibly due to the saturation of the binding between the immobilized L10-2 and soluble SARS-CoV-2 S-protein.

**Figure 5:**
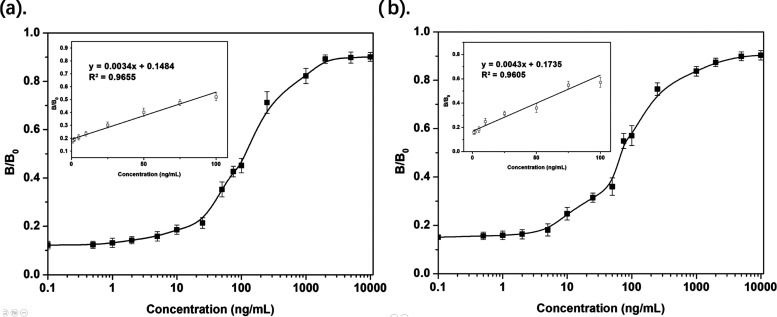
Corresponding *B*/*B*_0_ value of (a) Delta-variant SARS-CoV-2 spike proteins and (b) saliva spiked with wild-type SARS-CoV-2 spike proteins over a range of concentrations.

Using a camera to extract red (R), green (G), and blue (B) values of the color intensity change from biosensors can lead to better qualitative and quantitative analysis of the presence of the S-protein. We acquired normalized *R*-value changes (*B*/*B*_0_) by a smartphone camera and plotted the changes against S-protein concentrations (Figure 4b). The plot revealed a linear working range between 1 and 100 ng/mL S-protein, and the limit of detection (LOD) of the biosensor is 18.3 ng/mL (Figure 4c). Furthermore, as the BLI experiments (Supporting Information S3.2) also reveal that L10-2 can bind to S-protein from the SARS-CoV-2 Delta variant, we also tested the L10-2-modified nanofibrous biosensor for Delta-variant S-protein detection. We found the performance of the biosensor toward the S-protein from the Delta variant is comparable to that of the wild-type S-protein, with a comparable working range and LOD where the difference between the 1 ng/mL sample and the negative control group can be distinguished from the analysis of pictures taken by the smartphone (Figure 5a).

We further performed experiments to investigate the performance of the biosensor in saliva. We diluted the SARS-CoV-2 S-protein stock solution with human saliva for detection. To improve the observation of the SARS-CoV-2 S-protein in human saliva, sample preparation through centrifugation was employed to remove irrelevant biological substances or impurities which may result in a higher background signal. After centrifugation, the difference between 2 ppb and the negative control group was significant (Figure 5b). The LOD of the assay in saliva was 28.9 ng/mL, which was slightly higher than the pure protein due to the fact that the mixture of different proteins existing in saliva reduced the diffusion of the S-protein inside the nanofibrous membrane material, consequently lowering the sensitivity of the biosensor.

Compared to current rapid on-site testing, the nanofibrous membrane-based biosensor using the peptide as the S-protein capturing ligand exhibited comparative advantages in the sensitivity of naked-eye distinction (Table S8).

## Conclusions

Several peptidic ligands against the SARS-CoV-2 S-protein were discovered and optimized with OBOC combinatorial technology using enzyme-linked colorimetric screening and homolog library design. The peptide with the highest binding affinity, L10-2, when immobilized on the microporous nanofibrous membrane, affords an inexpensive and robust biosensor with high sensitivity for naked-eye detection of the S-protein at low ng/mL levels in saliva samples for both the wild-type and Delta variant. This technology platform can potentially be utilized for the rapid development of sensitive, economical, and rapid household diagnostics against microbes of future epidemics and pandemics. The fact that L10-2 can bind both wild-type and Delta-variant S-proteins suggests that it could be an excellent lead compound for the development of novel therapeutics against COVID-19. Furthermore, we have demonstrated how OBOC can be employed to rapidly discover peptidic ligands for detection of viral proteins. This strategy, when combined with an appropriate nanoscaffold, can be readily used to develop biosensors for other viruses in the future with robuustness to overcome rapid mutations.

## Supporting Information Available

OBOC library design, lead optimization, BLI assay, characterization of nanofibrous modification, specificity of the assay, and MALDI-TOF of peptidic ligands (PDF)
