# Supplementary material for: Discovery of Peptidic Ligands against the SARS-CoV-2 Spike Protein and Their Use in the Development of a Highly Sensitive Personal Use Colorimetric COVID-19 Biosensor
Source: ACS Sens. 2023 May 30;8(6):2159–68. doi: 10.1021/acssensors.2c02386 (PMC10255569; doi:10.1021/acssensors.2c02386)
Supplement: Supplementary file 1 — se2c02386_si_001.pdf [file se2c02386_si_001.pdf]

Supporting Information

Discovery of Peptidic Ligands against the SARS-CoV-2 Spike Protein and Their Use in the Development of a Highly Sensitive Personal Use Colorimetric COVID-19 Biosensor

Xingjian Yu[a,c], Bofeng Pan[b], Cunyi Zhao[b], Diedra Shorty[a,c], Lucas N. Solano[a], Gang Sun[b]*, Ruiwu Liu[a]*, and Kit S. Lam[a]*

[a] Department of Biochemistry & Molecular Medicine, University of California, Sacramento, CA 95817, USA

[b] Department of Biological and Agricultural Engineering, University of California, Davis, CA, 95616, USA

[c] Department of Chemistry, University of California, Sacramento, CA 95616, USA

Table of Content

S1 Random OBOC Combinatorial Library Design for Initial Screening

S2 Focused OBOC Library Design for Lead Optimization

S3 Kinetics Studies and Binding Affinity Characterization of SARS-CoV-2 Spike Protein Binding Peptides by Streptavidin-based Biolayer Interferometry (BLI) Assay

S4 Characterization of Nanofibrous Membrane

S5 Immobilization of Spike Protein Binding Peptides onto the Nanofibrous Membrane

S6 Specificity of the SARS-CoV-2 Spike Protein assay

S7 Mass Spectrometry of Biotinylated SARS-CoV-2 Spike Protein Binding Peptides

S8 Comparison Table

S1 Random OBOC Combinatorial Library Design for Initial Screening

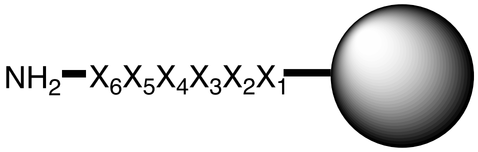

Figure S1 Design of 6-AA Linear OBOC Libraries

Table S1 Amino Acid Composition for Random 6AA Linear Library

Fmoc-Ala-OH<br>A<br>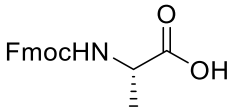	Fmoc-Arg(Pmc)-OH<br>R<br>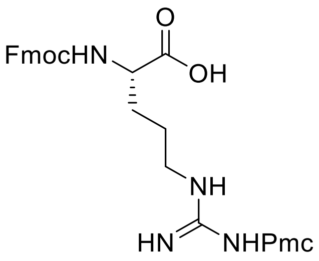	Fmoc-Asn(Trt)-OH<br>N<br>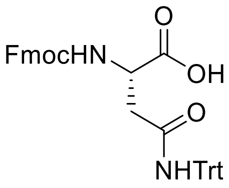	Fmoc-Asp(tBu)-OH<br>D<br>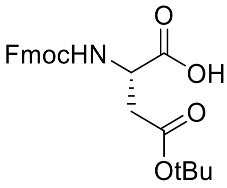
Fmoc-Gln(Trt)-OH<br>Q<br>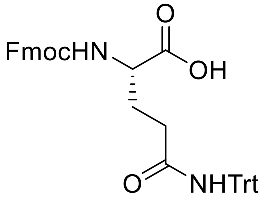	Fmoc-Glu(tBu)-OH<br>E<br>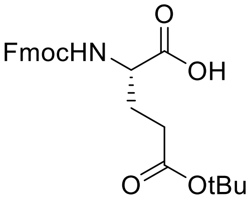	Fmoc-Gly-OH<br>G<br>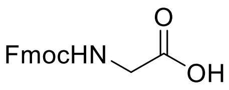	Fmoc-His(Trt)-OH<br>H<br>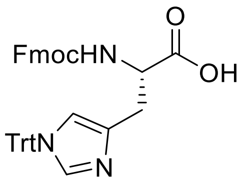
Fmoc-Leu-OH<br>L<br>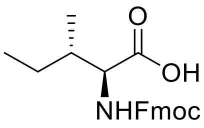	Fmoc-Ile-OH<br>I<br>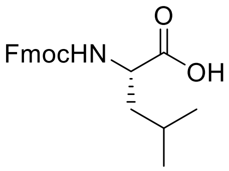	Fmoc-Met-OH<br>M<br>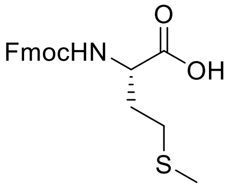	Fmoc-Phe-OH<br>F<br>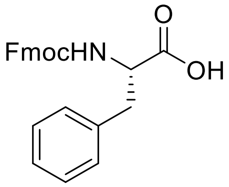
Fmoc-Pro-OH<br>P<br>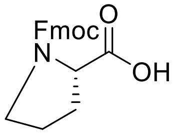	Fmoc-Ser(tBu)-OH<br>S<br>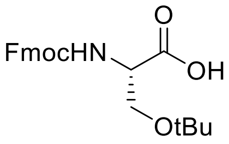	Fmoc-Thr(tBu)-OH<br>T<br>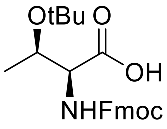	Fmoc-Trp(Boc)-OH<br>W<br>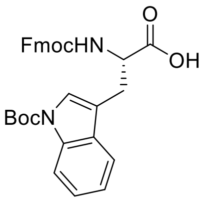

Fmoc-Tyr(tBu)-OH<br>Y<br>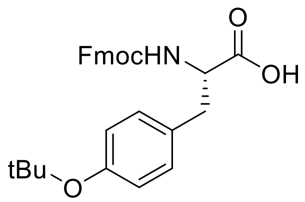	Fmoc-Val-OH<br>V<br>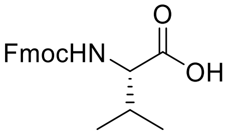	Fmoc-Hyp(tBu)-OH<br>Hyp<br>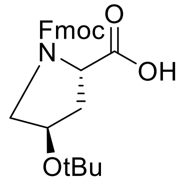	Fmoc-3-Pal-OH<br>3-Pal<br>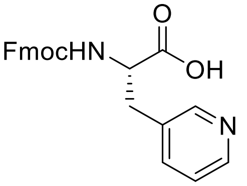
Fmoc-Aib-OH<br>Aib<br>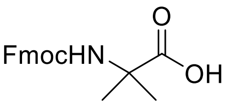	Fmoc-Nva-OH<br>Nva<br>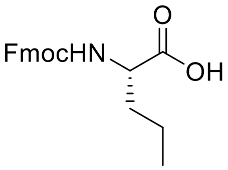	Fmoc-Dpr(Boc)-OH<br>Dpr<br>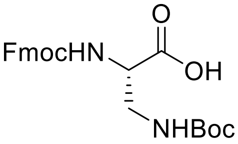	Fmoc-Tyr(Me)-OH<br>Tyr(Me)<br>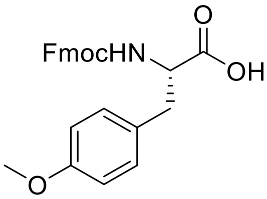
Fmoc-Nle-OH<br>Nle<br>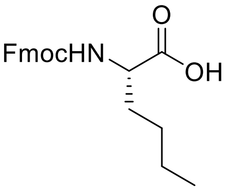	Fmoc-Phe(4-Me)-OH<br>4-Me<br>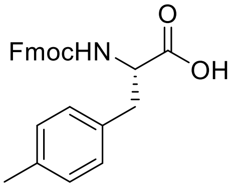	Fmoc-Cha-OH<br>Cha<br>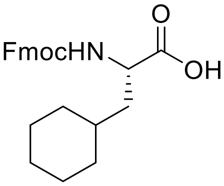	Fmoc-Chg-OH<br>Chg<br>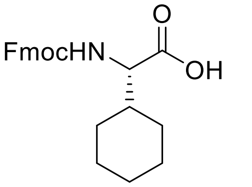
Fmoc-Phe(3,5-diF)-OH<br>3,5-diF<br>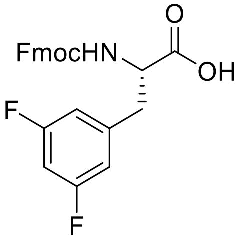	Fmoc-Aad(tBu)-OH<br>Aad<br>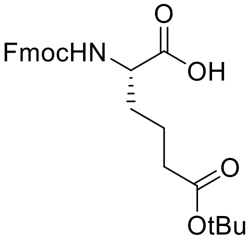		

Table S2 SARS-CoV-2 Affinity Peptides Discovered from the Random 6-mer Linear Peptide Library

	X6	X5	X4	X3	X2	X1
**COV6-1**	Acpc	T	M	G	Phe(3,5-diF)	Dpr
**COV6-2**	R	Q	Tyr(Me)	F	Phe(3,5-diF)	D
**COV6-3**	I	R	Tyr(Me)	L	Hyp	H
**COV6-4**	M	R	L	V	A	I
**COV6-5**	E	R	T	R	Dpr	P

S2 Focused OBOC Library Design for Lead Optimization

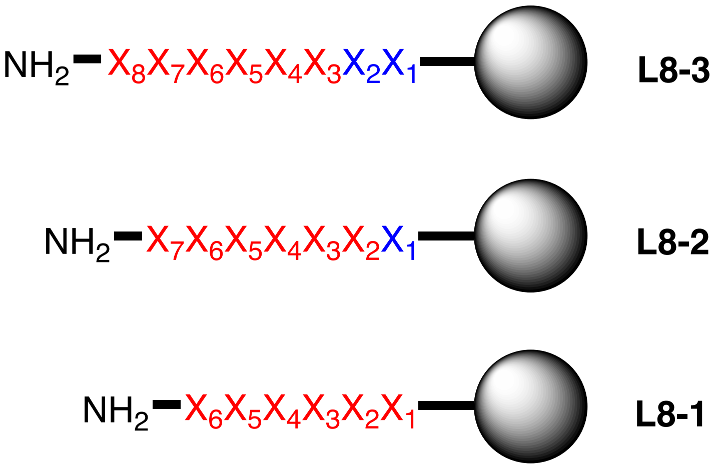

Figure S2 Linear 8-mer Peptide Libraries (L8); Red: Focused Sequence; Blue: Random Sequence

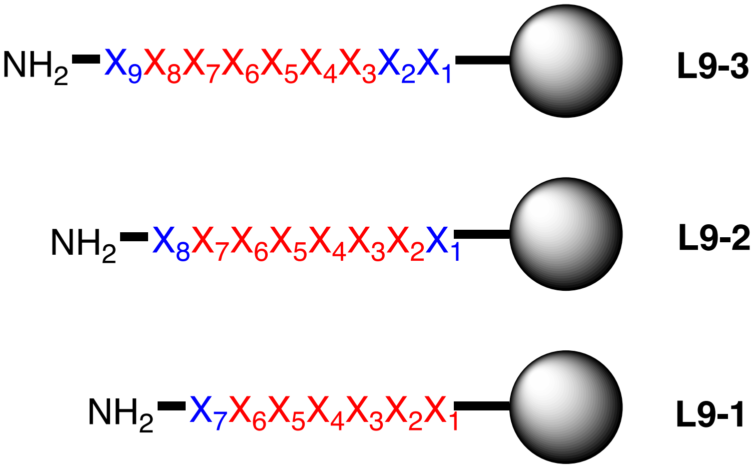

Figure S3 Linear 9-AA Libraries (L9); Red: Focused Sequence; Blue: Random residue

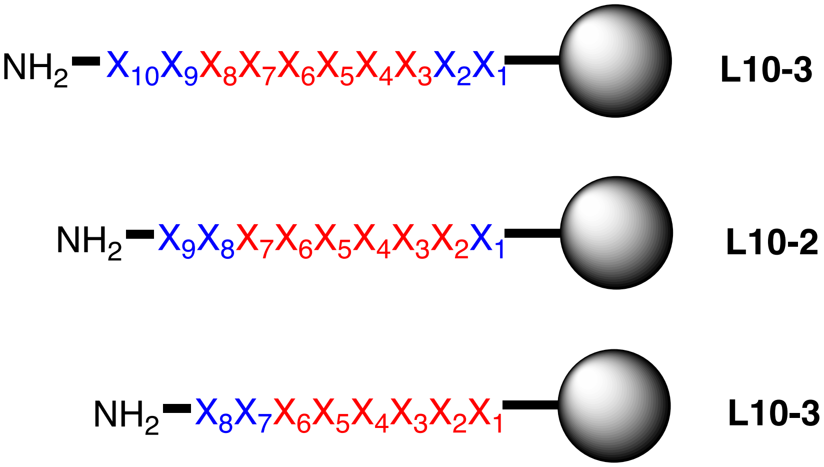

Figure S4 Linear 10-mer Peptide Libraries (L10); Red: Focused Sequence; Blue: Random Residues

Table S3 Amino Acid Composition of Focused OBOC Libraries L8-L10

X_{10}	X_9	X_8	X_7	X_6	X_5	X_4	X_3	X_2	X_1
33 aa	33 aa	R 20%	R 20%	AA in X5; Q, n, L-HoCit	Acpc, Aib, Tyr(Me), Ach, D-Nle, Phe (3,5 diF), D-Phg, Hyp, Nva, D-3-Pal,Phe (3-Cl), Phe (3,4 diCl), a, y, p, m,v,w,F,I,L,G	Hyp 20%	D 20%	33 aa	33 aa
		I 20%	Q 20%			Phe(di-F) 20%	h 20%		
		Rest 31 aa 60%	Rest 31 aa 60%			p 10%	e 10%		
					R, e, h,	Rest 30 aa 50%	Aad 10%, R10%, Rest 28 aa 30%		

Table S4 Amino Acid Composition for Focused Library for Lead Optimization

Fmoc-D-Ala-OH<br>a<br>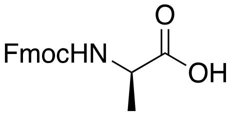	Fmoc-Arg(Pbf)-OH<br>R<br>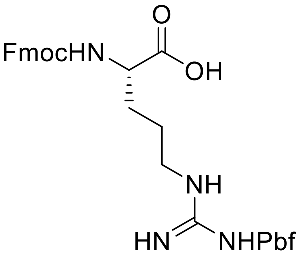	Fmoc-D-Asn(Trt)-OH<br>n<br>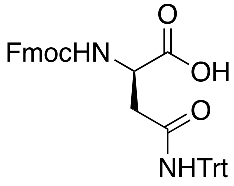	Fmoc-Asp(tBu)-OH<br>D<br>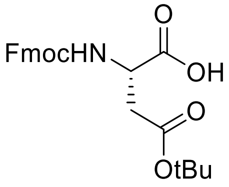
Fmoc-Gln(Trt)-OH<br>Q<br>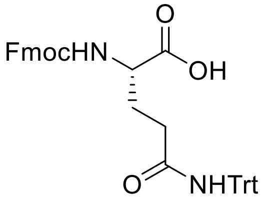	Fmoc-D-Glu(tBu)-OH<br>e<br>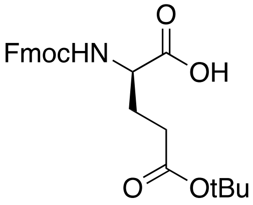	Fmoc-Gly-OH<br>G<br>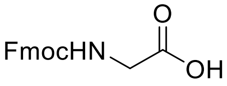	Fmoc-D-His(Trt)-OH<br>h<br>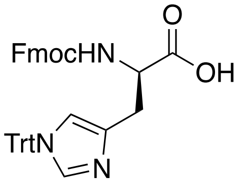
Fmoc-Leu-OH<br>L<br>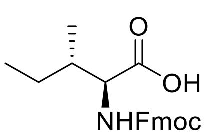	Fmoc-Ile-OH<br>I<br>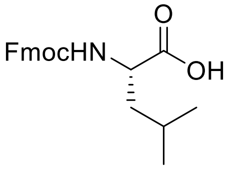	Fmoc-D-Met-OH<br>m<br>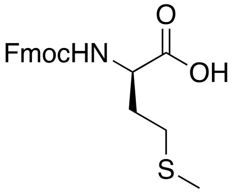	Fmoc-Phe-OH<br>F<br>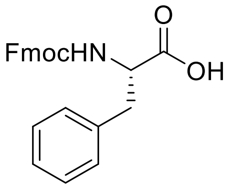
Fmoc-D-Pro-OH<br>p<br>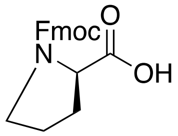	Fmoc-D-Ser(tBu)-OH<br>s<br>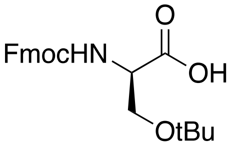	Fmoc-Thr(tBu)-OH<br>T<br>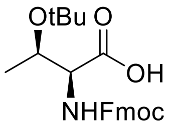	Fmoc-Trp(Boc)-OH<br>W<br>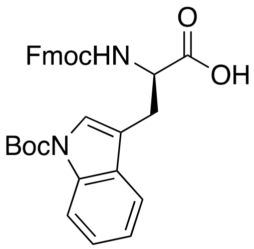
Fmoc-Tyr(tBu)-OH<br>Y	Fmoc-D-Val-OH<br>v<br>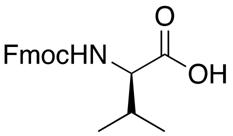	Fmoc-Hyp(tBu)-OH<br>Hyp<br>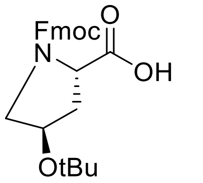	Fmoc-D-3-Pal-OH<br>D-3-Pal<br>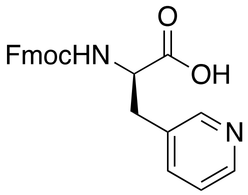

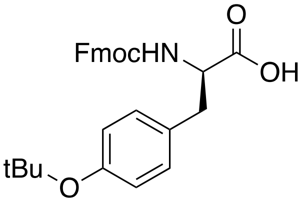			
Fmoc-Aib-OH<br>Aib<br>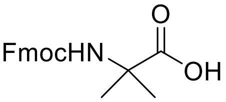	Fmoc-Nva-OH<br>Nva<br>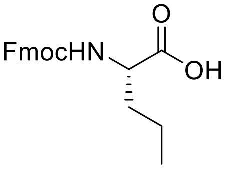	Fmoc-Acpc-OH<br>Acpc<br>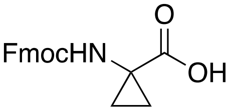	Fmoc-Tyr(Me)-OH<br>Tyr(Me)<br>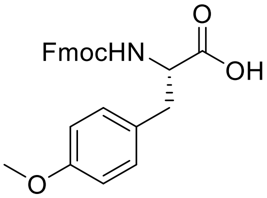
Fmoc-Nle-OH<br>Nle<br>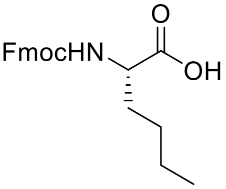	Fmoc-Phe(4-Me)-OH<br>4-Me<br>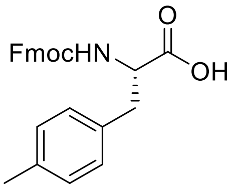	Fmoc-Cha-OH<br>Cha<br>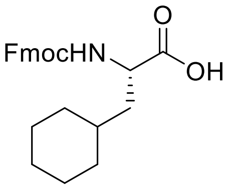	Fmoc-Chg-OH<br>Chg<br>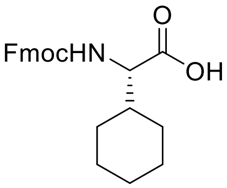
Fmoc-Phe(3,5-diF)-OH<br>3,5-diF<br>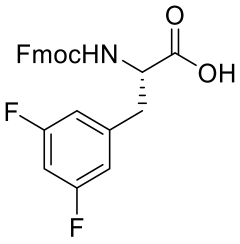	Fmoc-Aad(tBu)-OH<br>Aad<br>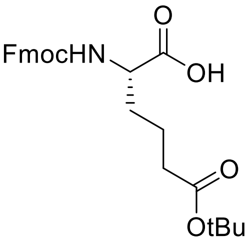	Fmoc-Phg-OH<br>Phg<br>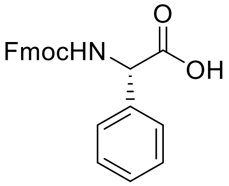	Fmoc-Phe(3,4-diCl)-<br>OH<br>3,4-diCl<br>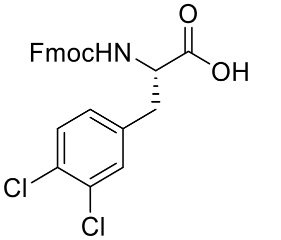
Fmoc-Aic-OH<br>Aic<br>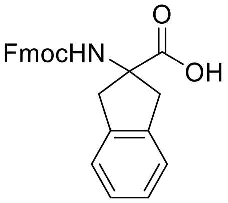	Fmoc-D-Phe(3-Cl)-OH<br>3-Cl<br>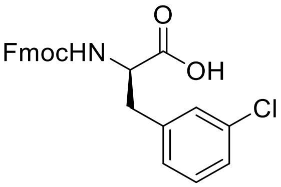	Fmoc-HoCit-OH<br>HoCit<br>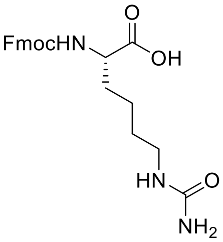	

Table S5 Peptides Discovered from Focused OBOC Libraries

	X10	X9	X8	X7	X6	X5	X4	X3	X2	X1
L9-1		Ach	m	HoCit	Nva	R	n	Phe(3,5-diF)	e	L
L10-3			Phe(3,5-diF)	Q	Nva	R	n	Nva	n	Acpc
L10-2	h	Tyr(Me)	R	HoCit	Nva	R	Phe(3,5-diF)	F	Aad	
L8-3	h	Tyr(Me)	R	D-Phg	D-3-Pal	Aad				
L8-1	h	Tyr(Me)	HoCit	D-Phg	e	m	h	L		
L10-4	h	Tyr(Me)	HoCit	Nva	R	Phe(3,5-diF)	F	Aad		

S3 Kinetics Studies and Binding Affinity Characterization of SARS-CoV-2 Spike Protein Binding Peptides by Streptavidin-based Biolayer Interferometry (BLI) Assay

S3.1 BLI Assay for SARS-CoV2 Spike Protein Trimer

Table S6 Statistics and Binding Affinity of Peptides Against Spike Protein Trimer Using BLI Assay

	Kd/M	RMax	R^2
CoV6-2	1.7×10^{-7}	0.85 ± 0.09	0.9483
L9-1	2.2×10^{-7}	0.91 ± 0.0031	0.9934
L10-2	2.5×10^{-8}	0.19 ± 0.012	0.9589
L10-3	2.0×10^{-7}	0.85 ± 0.0032	0.9916
L10-4	5.2×10^{-8}	0.24 ± 0.011	0.9890
L9-3	1.9×10^{-6}	1.29 ± 0.407	0.9872
Reference Peptide	7.9×10^{-8}	0.89 ± 0.014	0.9992

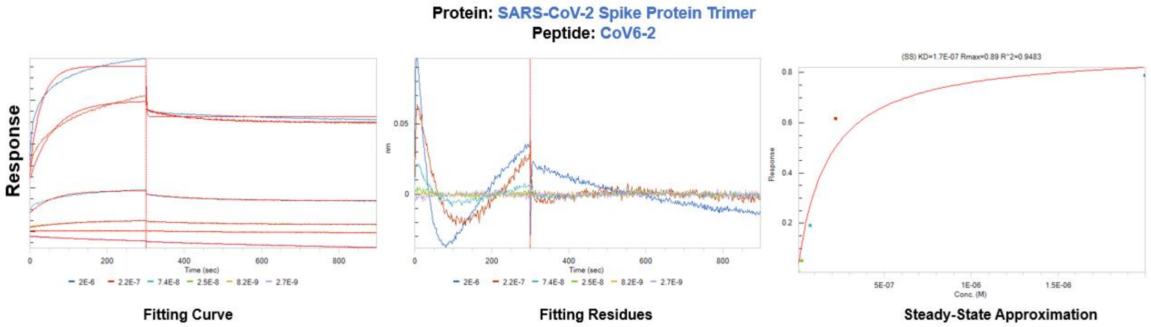

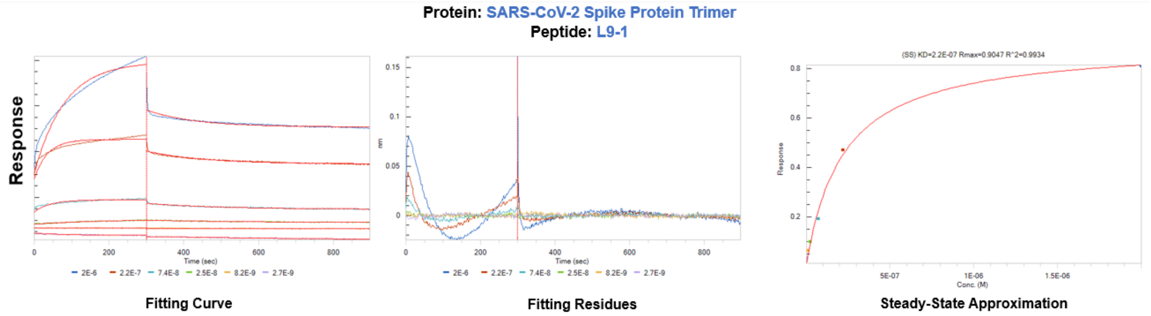

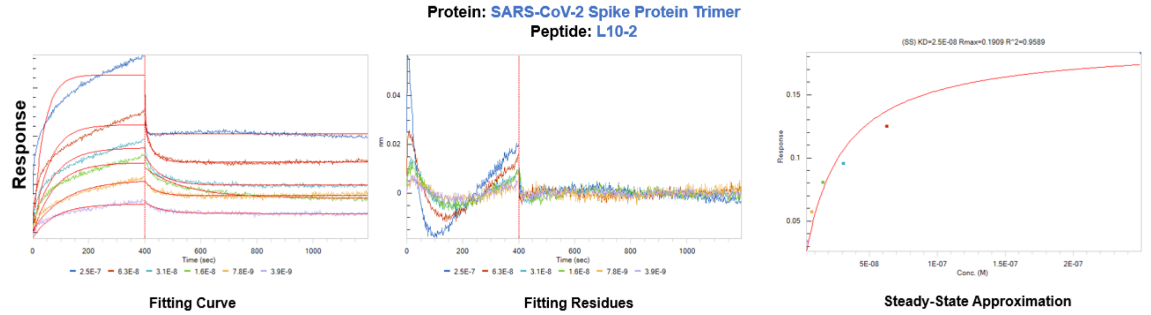

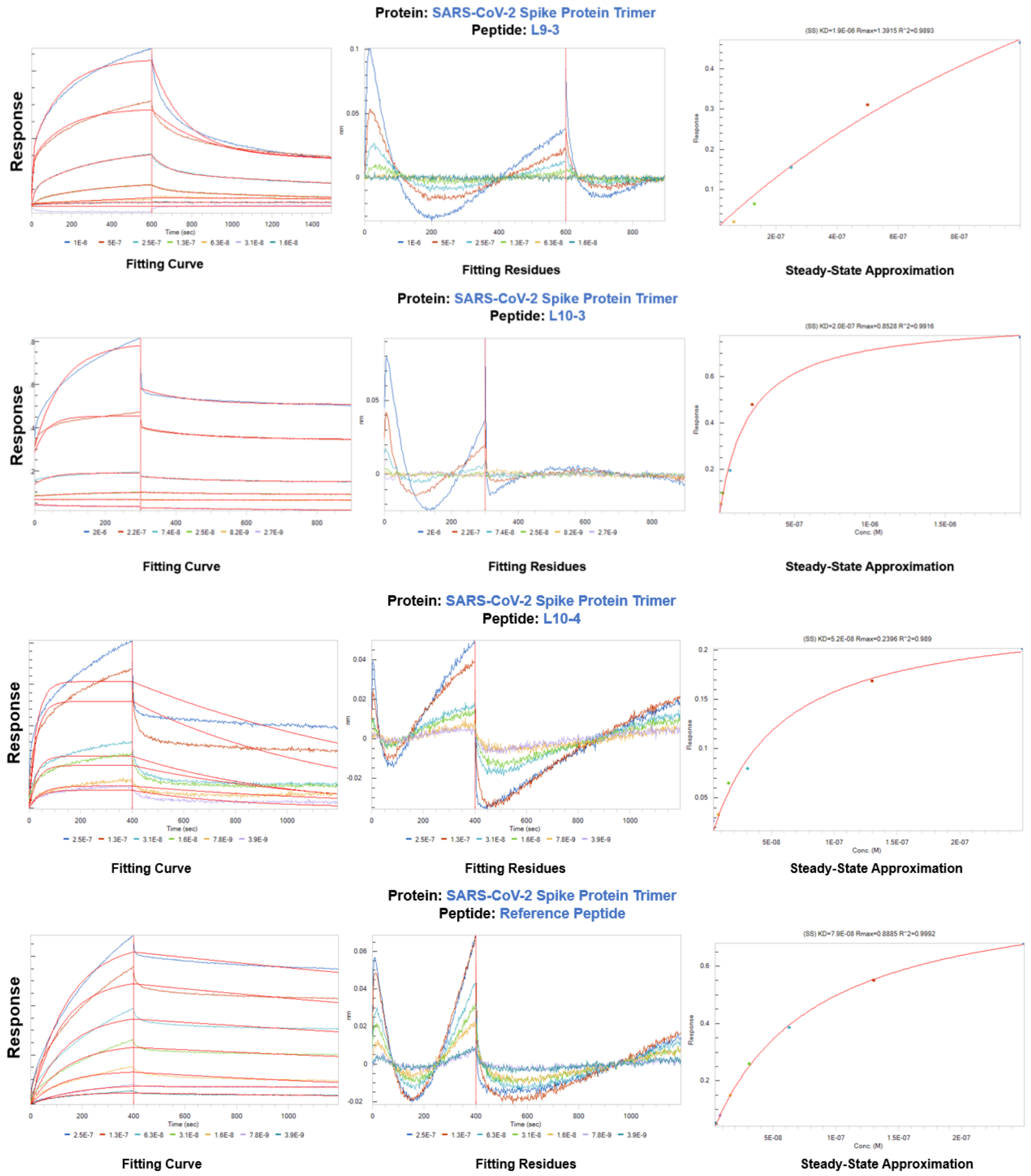

Figure S5 BLI Assay fitting curve, fitting resides, and steady state approximation for binding of peptide CoV6-2, L8-1, L9-1, L10-2, L10-3, and L10-4 against SARS-CoV-2 spike protein active trimer.

S3.2 BLI Assay for SARS-CoV2 Delta Variant Spike Protein RBD

Table S7 Statistics and Binding Affinity of Peptides Against Delta Variant Spike Protein Using BLI

	Kd/M	RMax	R^2
CoV6-2	2.5×10^{-7}	0.13 ± 0.003	0.9979
L10-2	2.7×10^{-7}	0.11 ± 0.004	0.9978

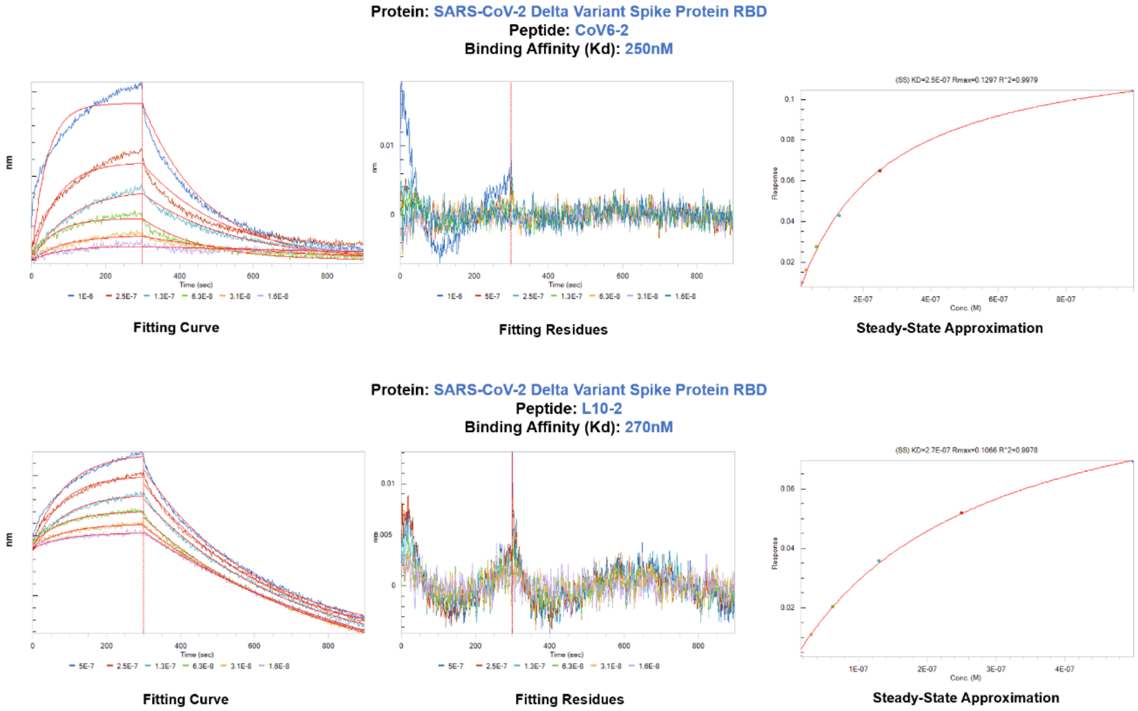

Figure S6 BLI Assay fitting curve, fitting resides, and steady state approximation for binding of peptide CoV6-2 and L10-2 against SARS-CoV-2 Delta Variant Spike Protein Receptor Binding Domain (RBD)

S4 Characterization of nanofibrous membrane

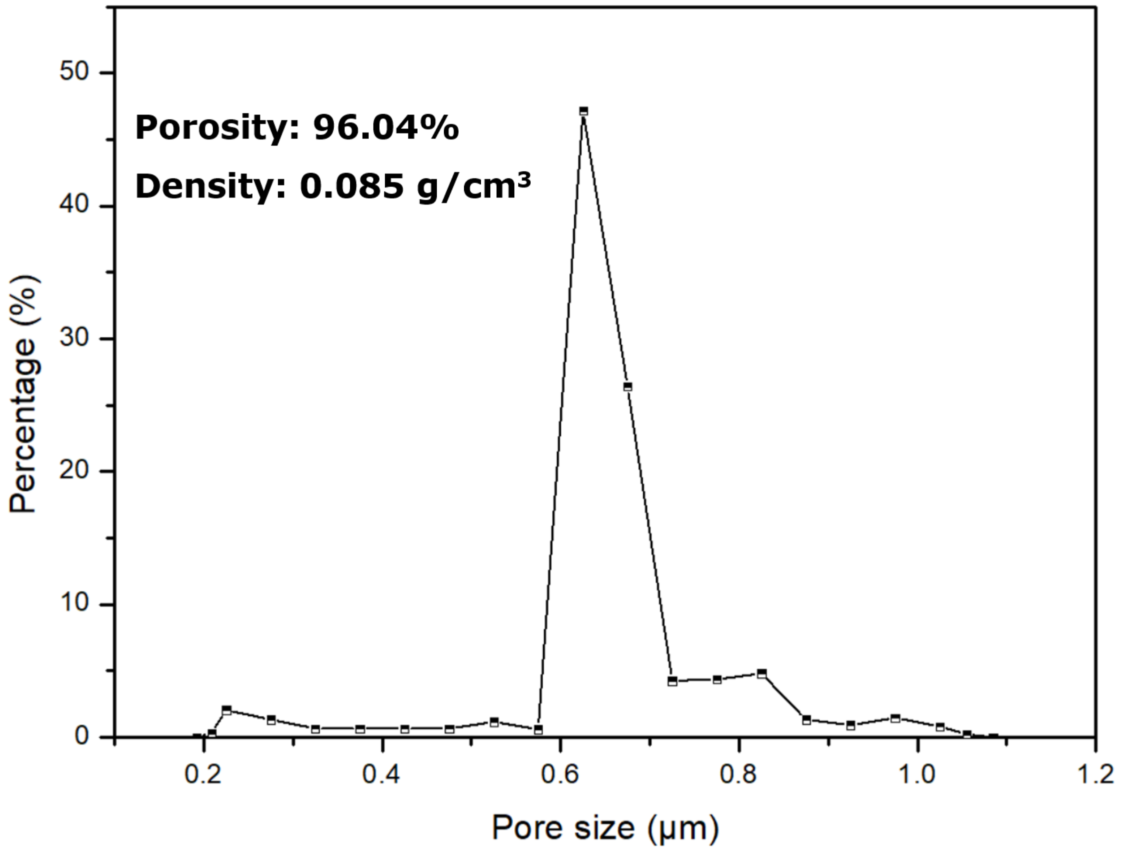

Figure S7. Density, porosity, and pore distribution of PVA-co-PE nanofibrous membrane.

S5 Immobilization of SARS-CoV-2 Spike Protein Peptides onto the Nanofibrous Membrane

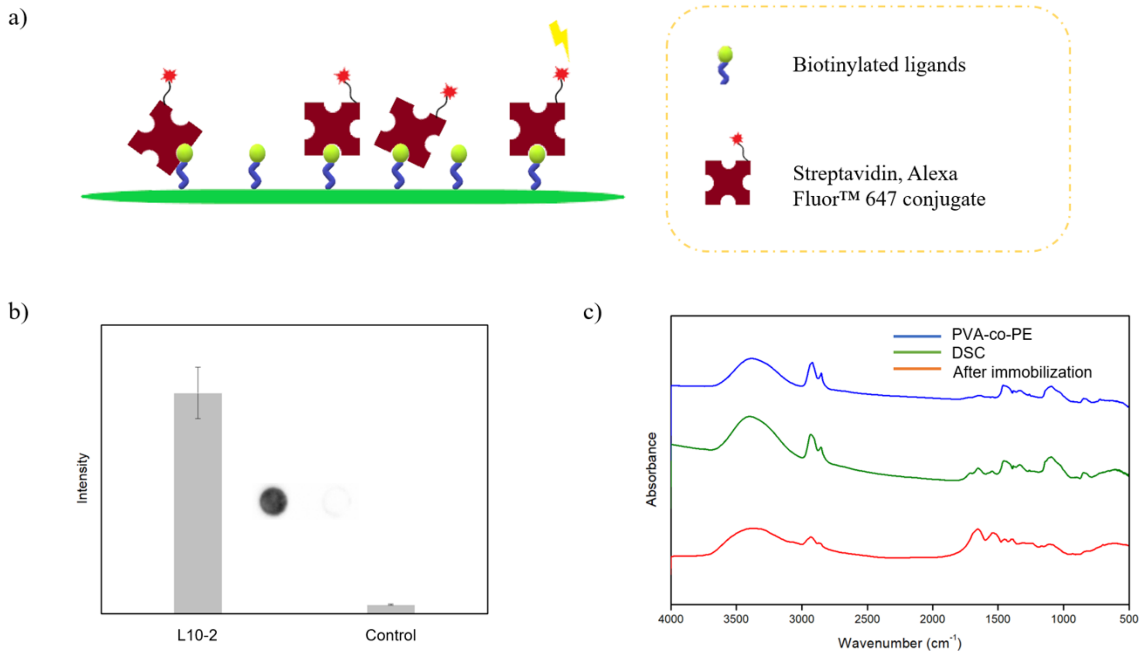

Figure S8. (a) Schematic illustration of the immobilization of peptide on DSC modified nanofibrous membrane; Biotinylated peptides were dissolved into PBS buffer and diluted to a concentration of 1mM. The DSC-modified nanofibrous membranes were exposed to a solution of L10-2 peptide under gentle agitation for 20 min. Then the active sites of the membranes were blocked by a 5% BSA solution. The membranes were then immersed into 0.25mg/mL streptavidin Alexa647 conjugate solution for 30min and were washed several times using the PBS buffer before measurement. The fluorescent intensity of the nanofibrous membrane was recorded by a microplate reader (SpectraMax iD3 multi-Mode). Bio-Rad imaging system was used to visualize the distribution of immobilized peptides on the membranes. (b) fluorescent signal intensity and image of nanofibrous membrane with immobilized L10-2 measured by the plate reader and Bio-Rad imaging system, respectively. (c) FTIR results of nanofibrous membranes (NFM) at different steps of peptide immobilization: original PVA-co-PE NFM, DSC modified NFM, and peptide immobilized NFM.

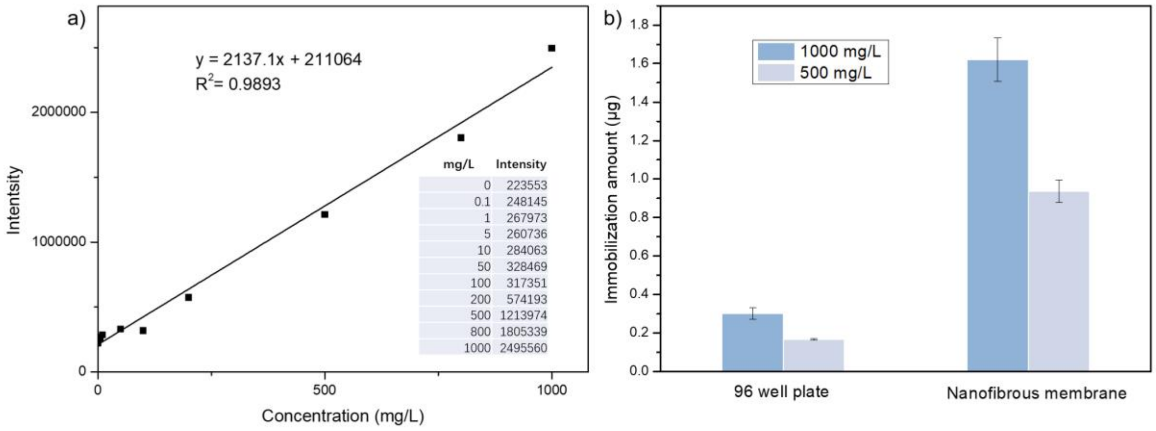

Figure S9. a) Calibration curve for peptide L10-2 using pierce quantitative fluorometric peptide assay. b) Immobilized peptide amounts on 96 well plate and nanofibrous membrane from 1000 mg/L and 500 mg/L of 100 µL of L10-2 solutions.

S6 Specificity and Performance of the SARS-CoV2 Spike Protein Assay.

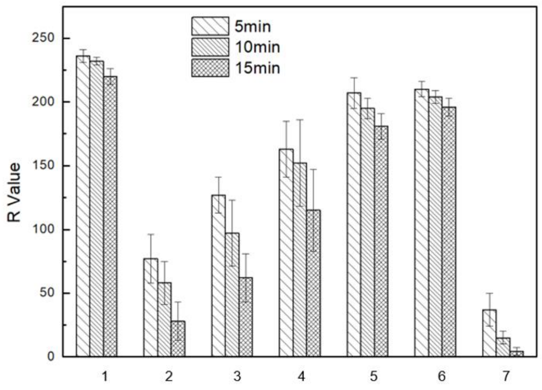

#	L10-2	SL10-2	BSA	S-Protein	Anti-SP-HRP
1	×	×	×	×	×
2	√	×	√	√	√
3	×	√	√	√	√
4	√	×	√	×	√
5	×	×	√	√	√
6	×	×	√	×	√
7	×	×	×	×	√

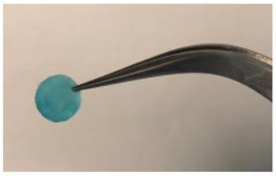

Figure S10. Specificity of the SARS-CoV2 spike protein assay. Colorimetric assay was performed on the NFM discs (photograph insert) under different experimental conditions as shown in the table. The bar diagram shows the R value observed at 5, 10, and 15 min under different conditions. The membrane discs were incubated with S-protein solution at 100 ng/mL. The unmodified nanofibrous membrane (no peptide) showed minimal non-specific interaction with S-protein, BSA, and antibodies (Exp.1, 5, 6). Additionally, it showed that the modification with scrambled L10-2 (SL10-2) peptides had a significantly lower color response than the sample group (Exp.3), which indicates the specificity of the peptide L10-2 to the spike protein. To obtain the relative qualitative signal for a specific concentration of the sample, the signal from the negative sample could be readily subtracted from the original data.

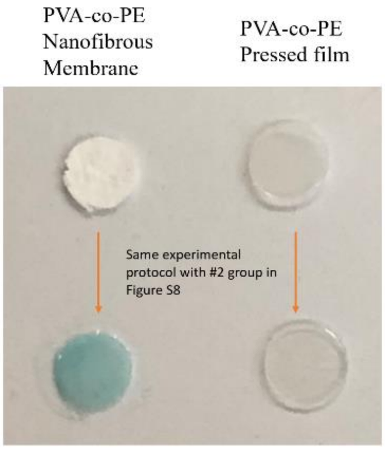

Figure S11. Comparison of capacity of nanofibrous membrane and non-nanofibrous membrane for affinity ligands.

S7 Chemical Structure and Mass Spectrometry (MALDI-TOF) of Biotinylated SARS-CoV-2 Spike Protein Binding Peptides

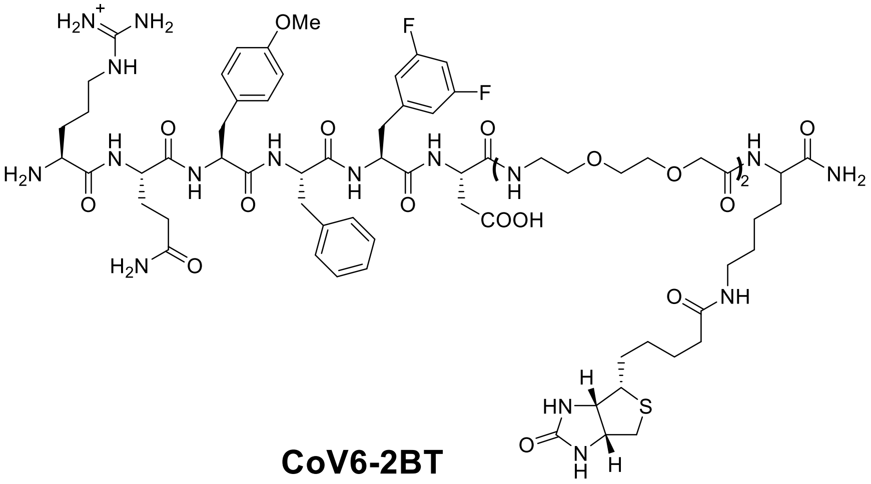

CoV6-2BT

Exact Mass: 1568.74

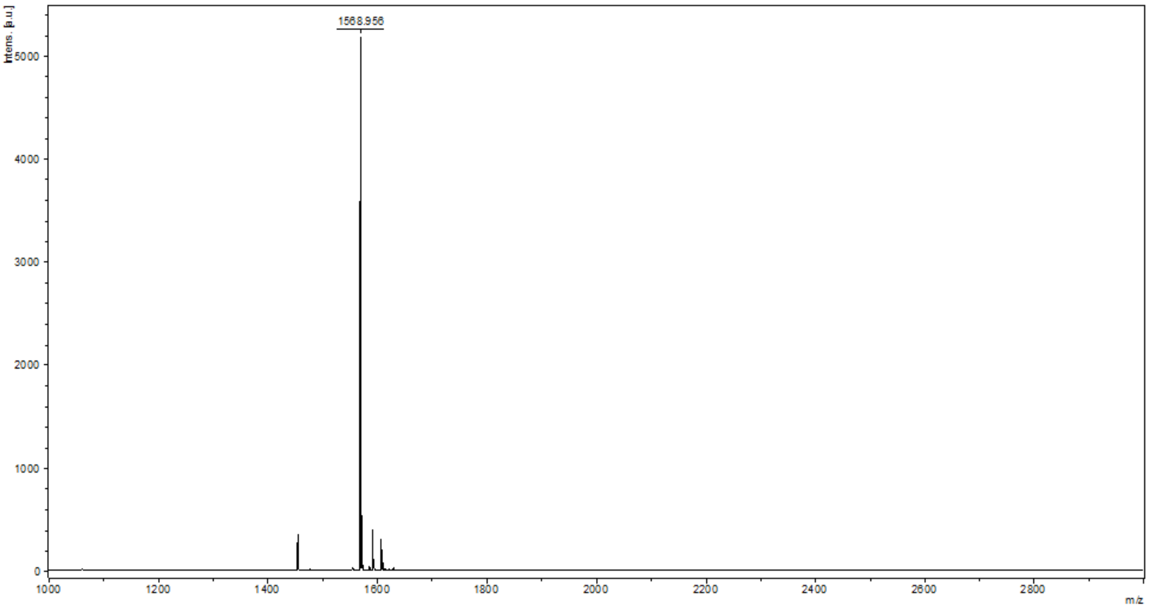

Figure S12 Chemical Structure and MALDI-TOF for Ligand CoV6-2BT

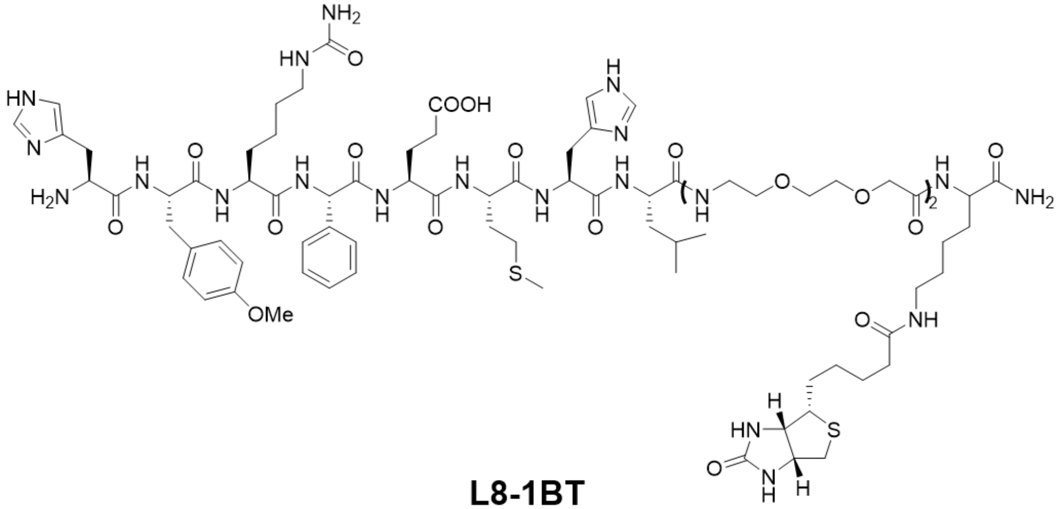

L8-1BT

Exact Mass: 1789.86

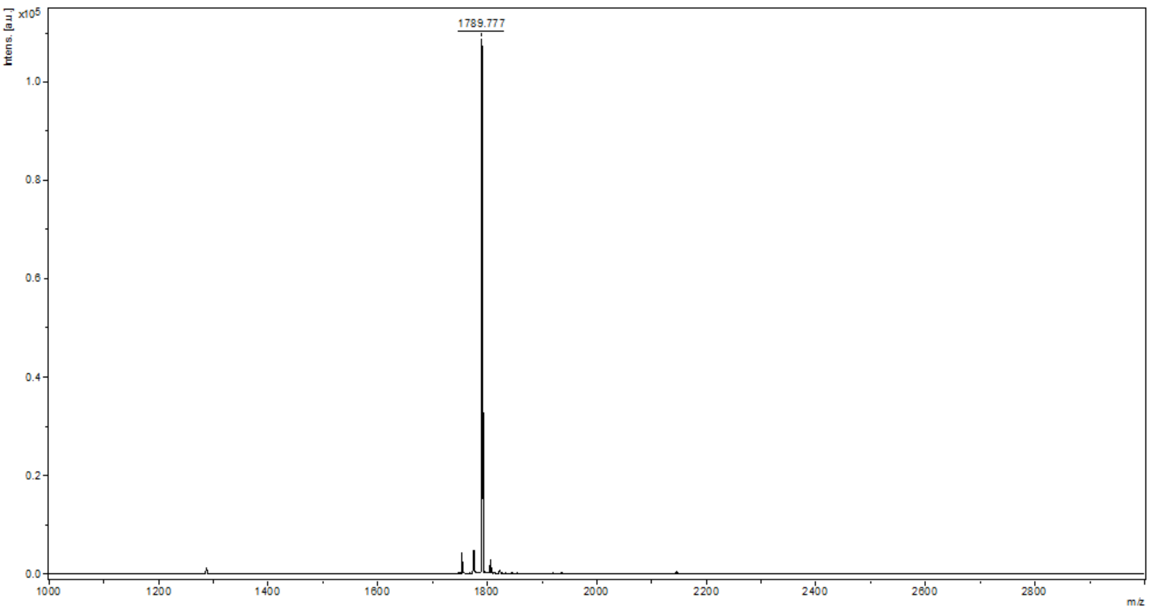

Figure S13 Chemical Structure and MALDI-TOF for Ligand L8-1BT

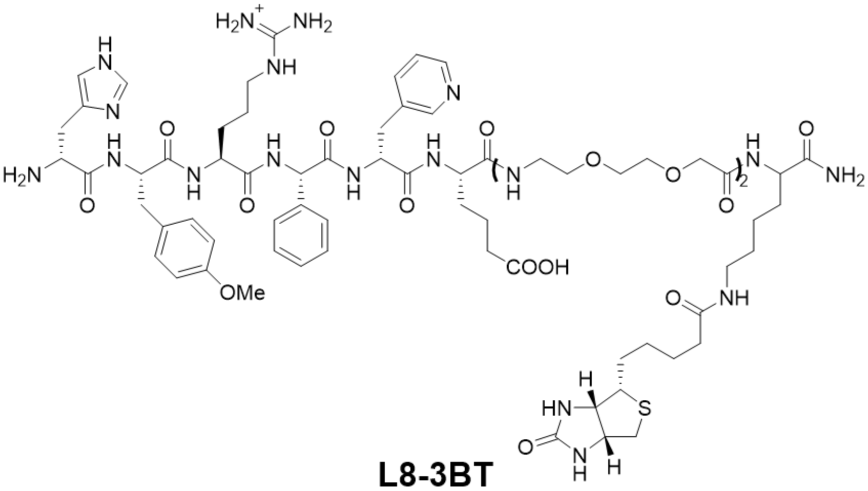

L8-3BT

Exact Mass: 1556.77

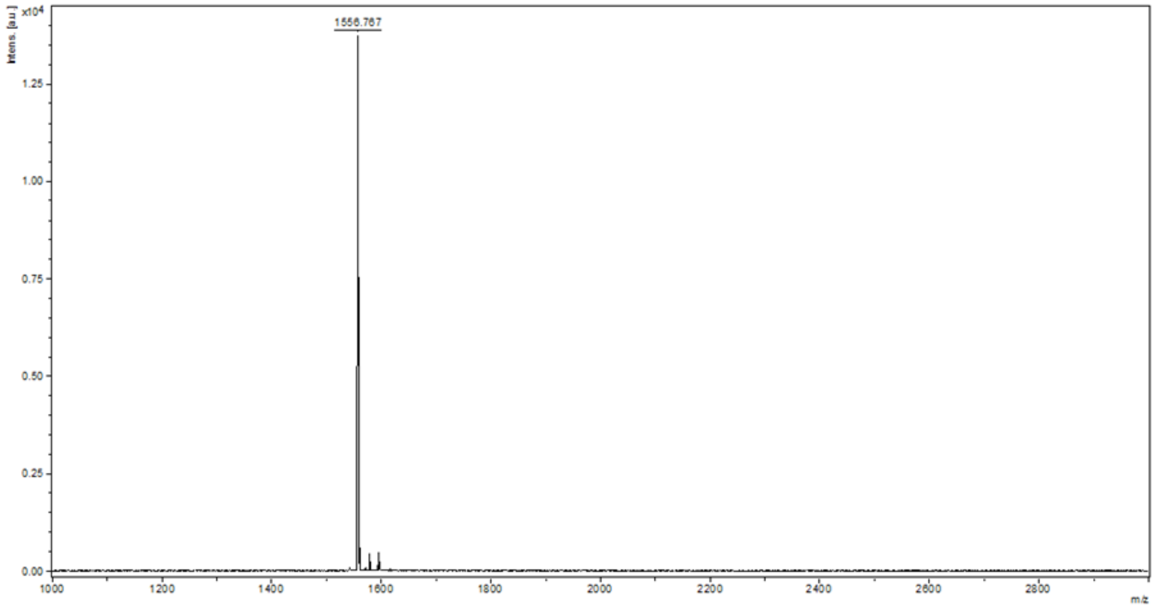

Figure S14 Chemical Structure and MALDI-TOF for L8-3BT

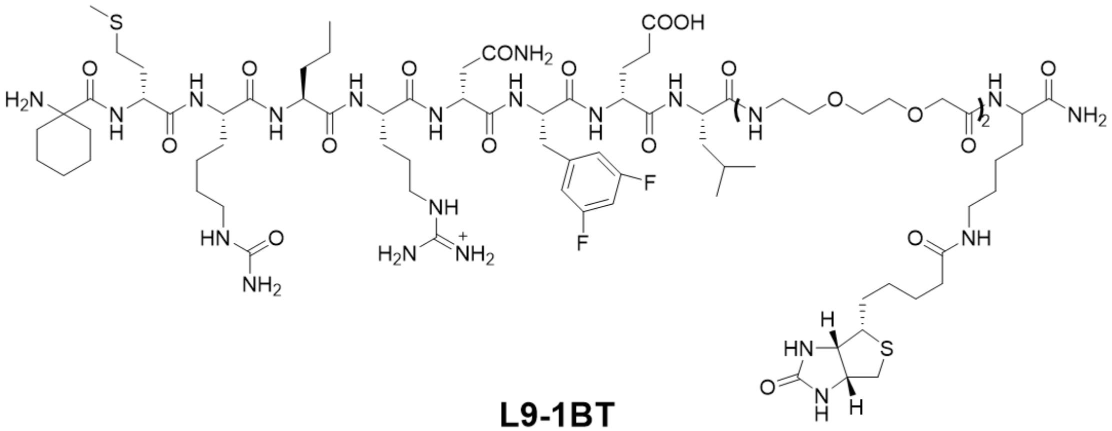

L9-1BT

Exact Mass: 1883.97

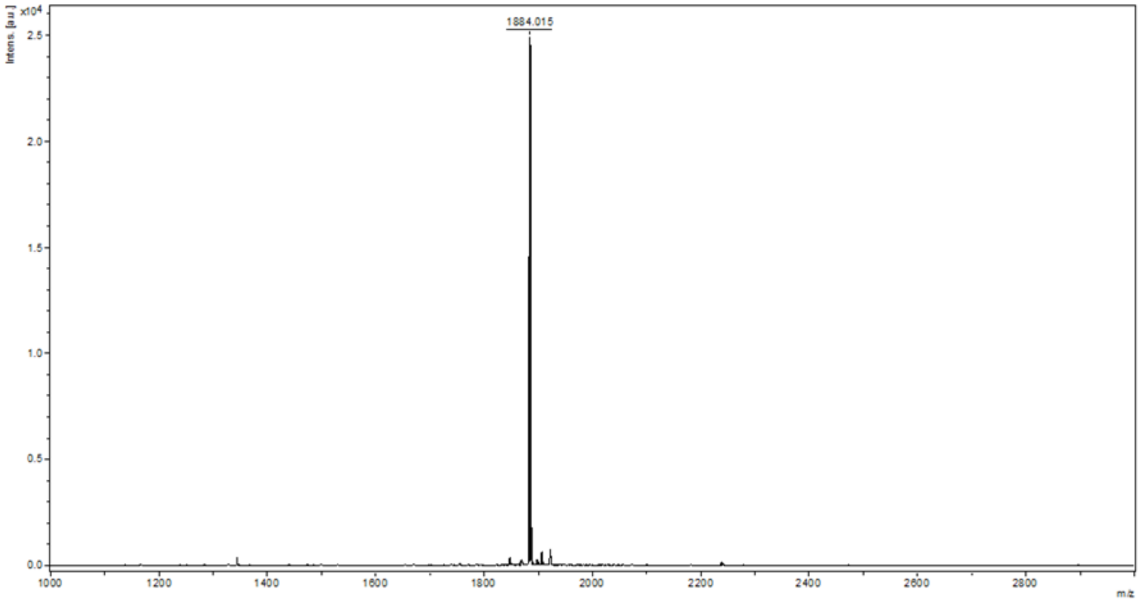

Figure S15 Chemical Structure and MALDI-TOF for Ligand L9-1BT

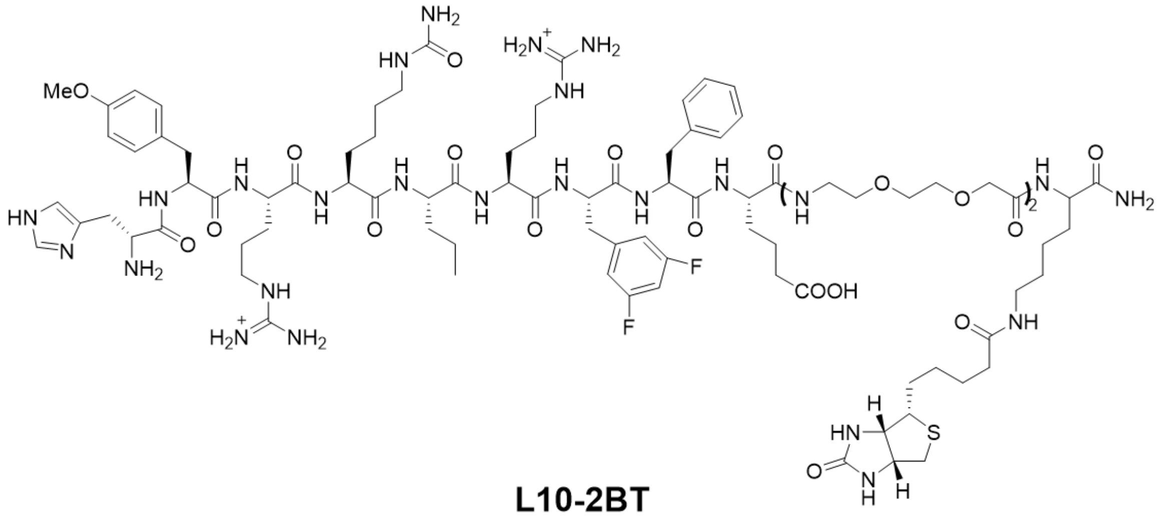

L10-2BT

Exact Mass: 2033.05

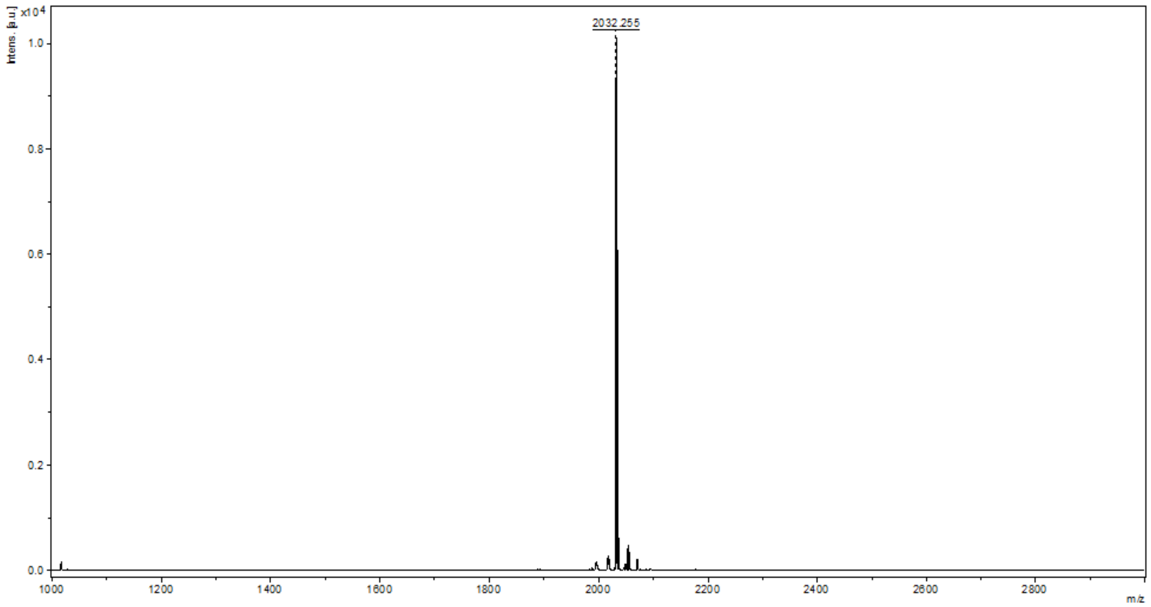

Figure S16 Chemical Structure and MALDI-TOF for Ligand L10-2BT

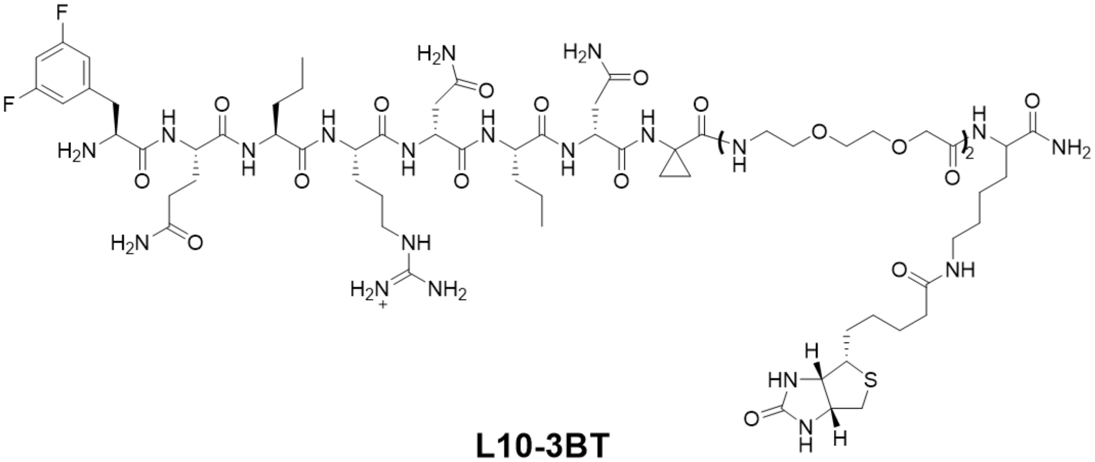

L10-3BT

Exact Mass: 1638.82

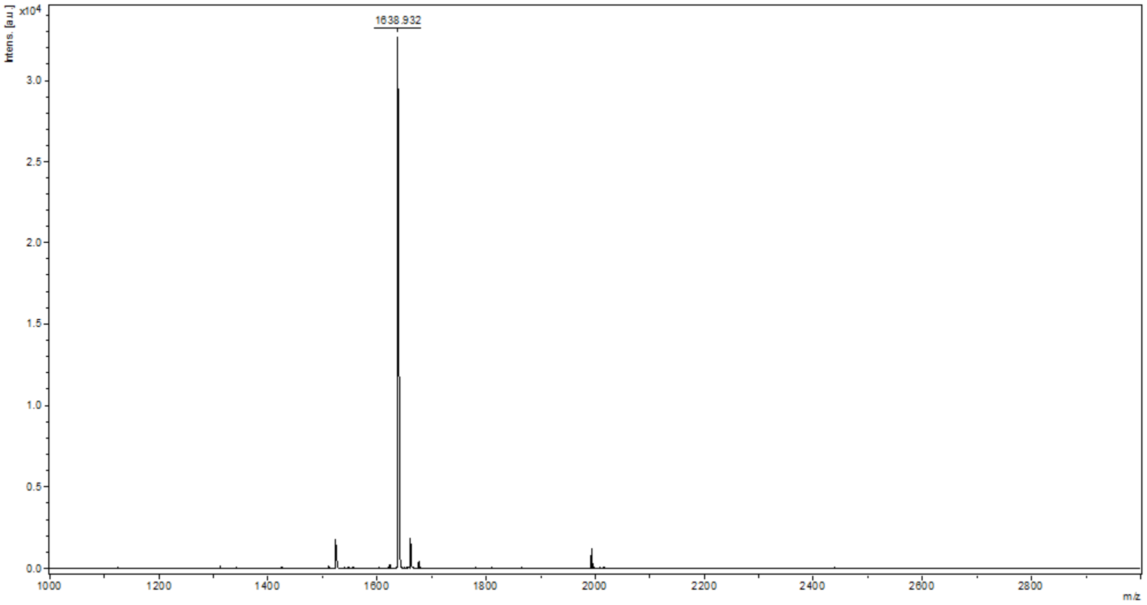

Figure S17 Chemical Structure and MALDI-TOF for L10-3BT

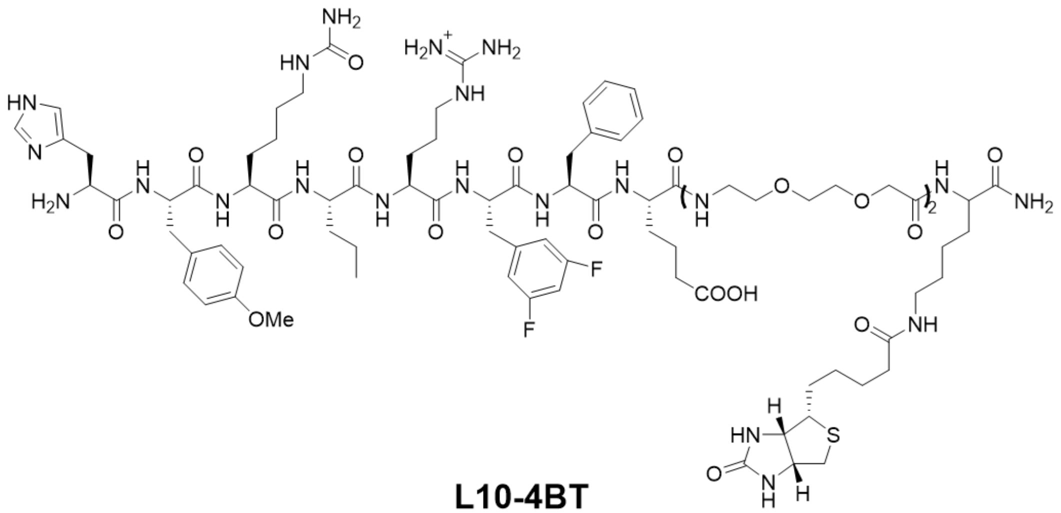

L10-4BT

Exact Mass: 1875.94

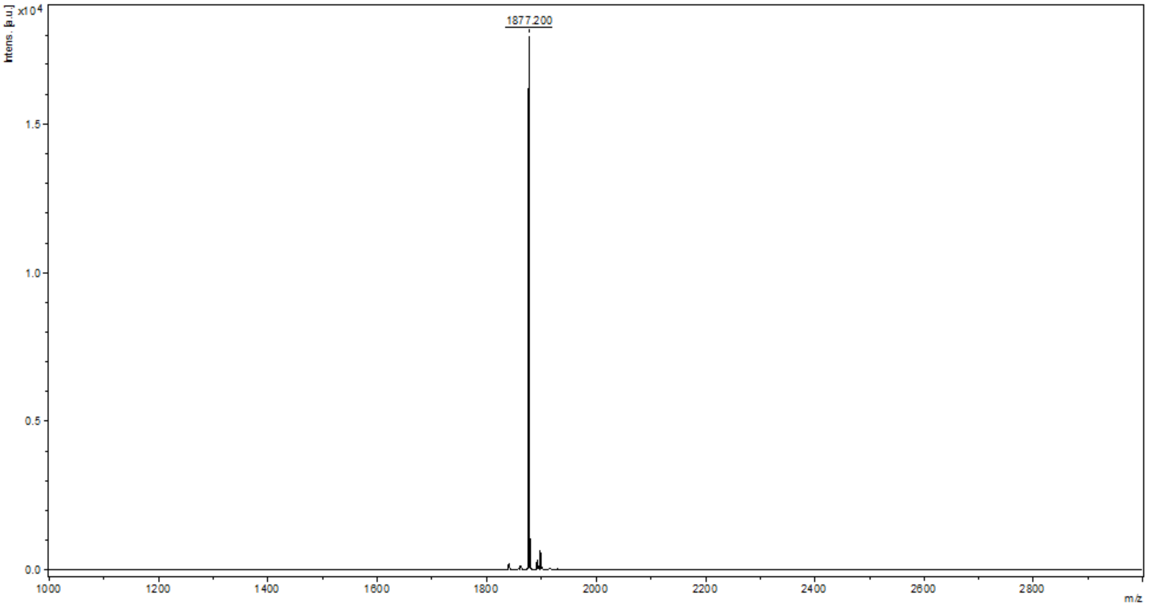

Figure S18 Chemical Structure and MALDI-TOF for L10-4BT

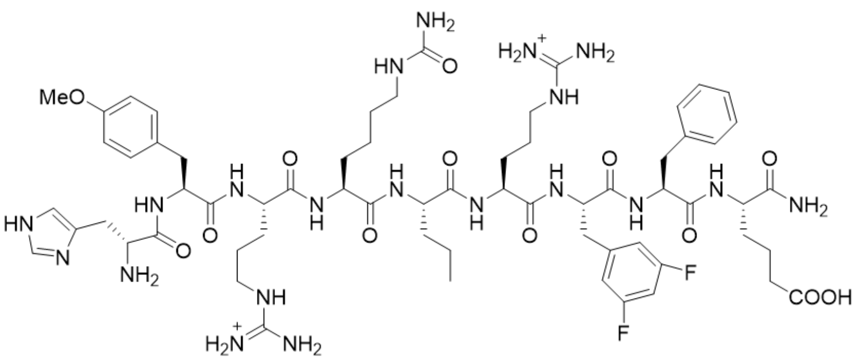

L10-2

Exact Mass: 1388.73

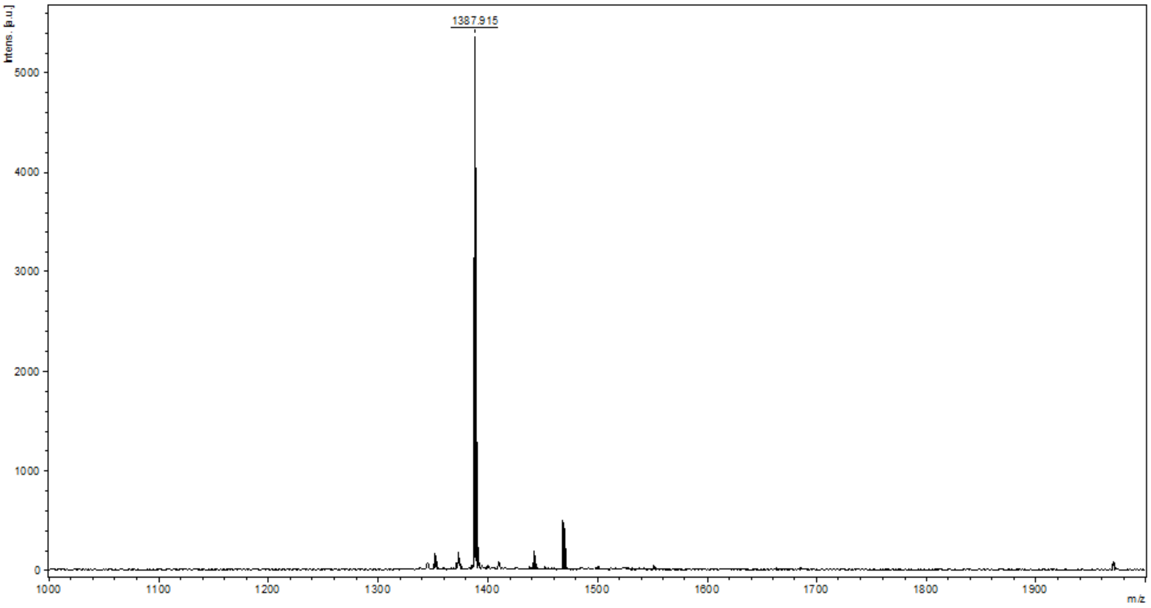

Figure S19 Chemical Structure and MALDI-TOF for L10-2

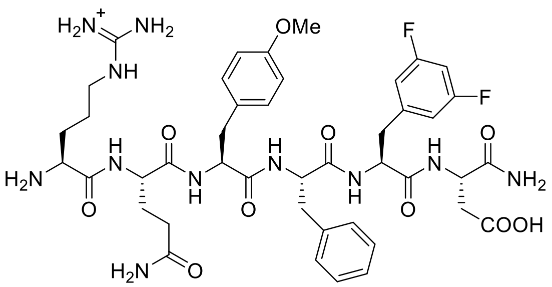

CoV6-2

Exact Mass: 924.42

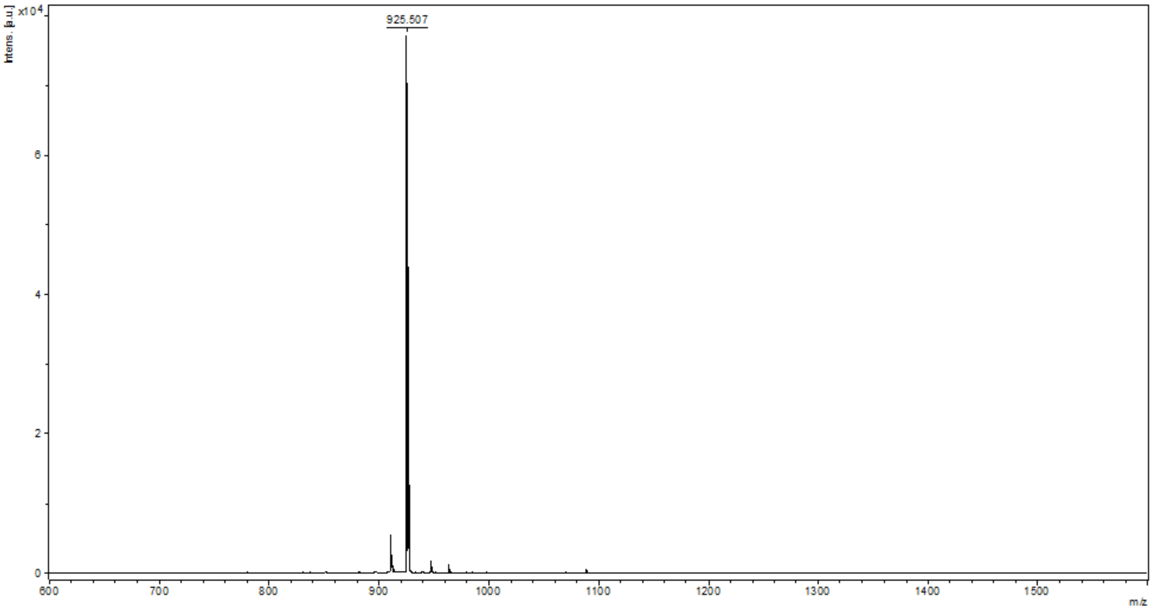

Figure S20 Chemical Structure and MALDI-TOF for L10-2

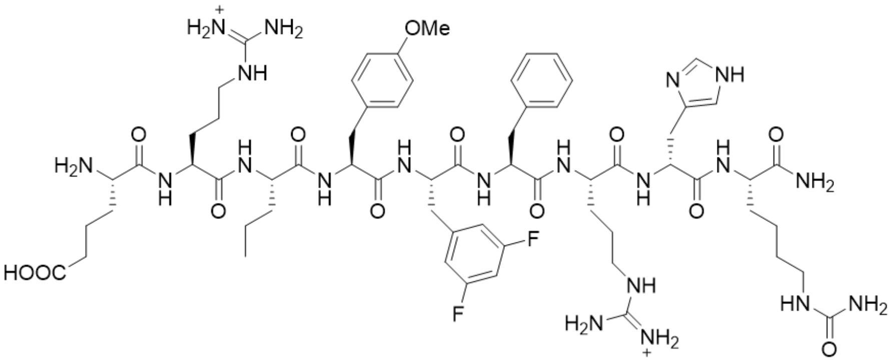

Exact Mass: 1388.73

Scramble-L10-2

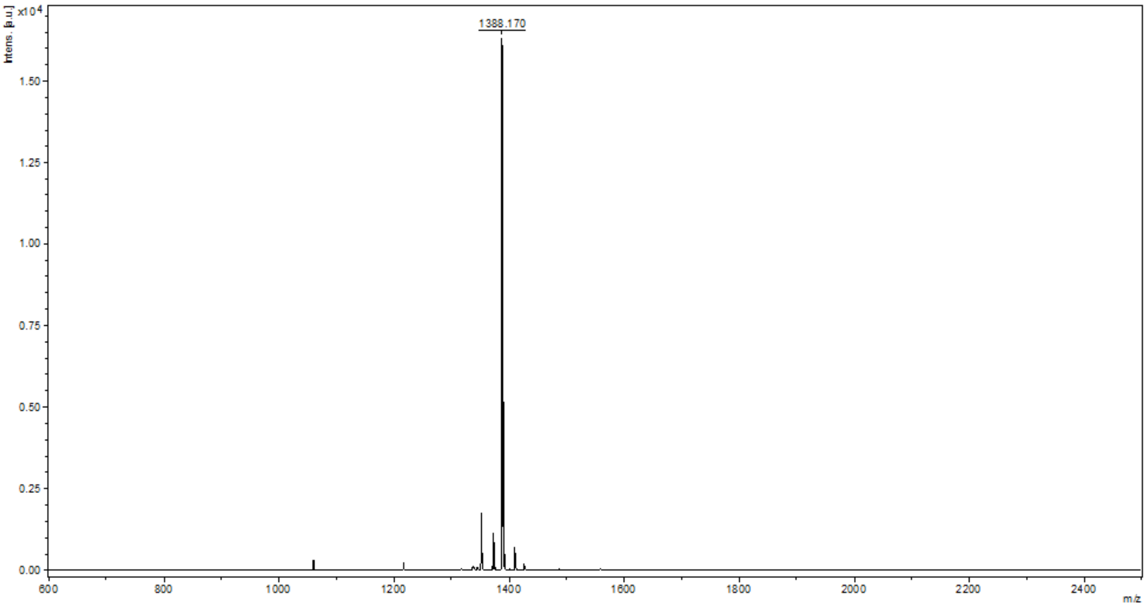

Figure S21 Chemical Structure and MALDI-TOF for Scrambled L10-2

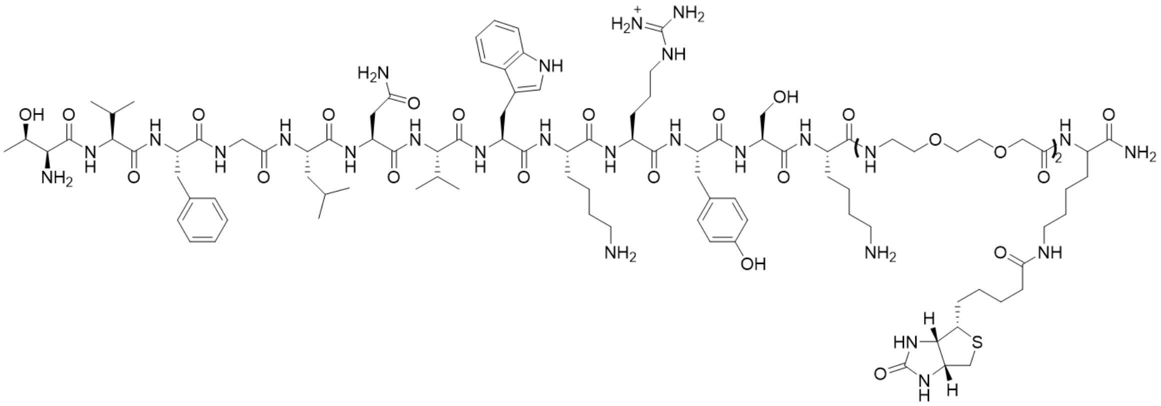

Reference Ligands

Exact Mass: 2241.22

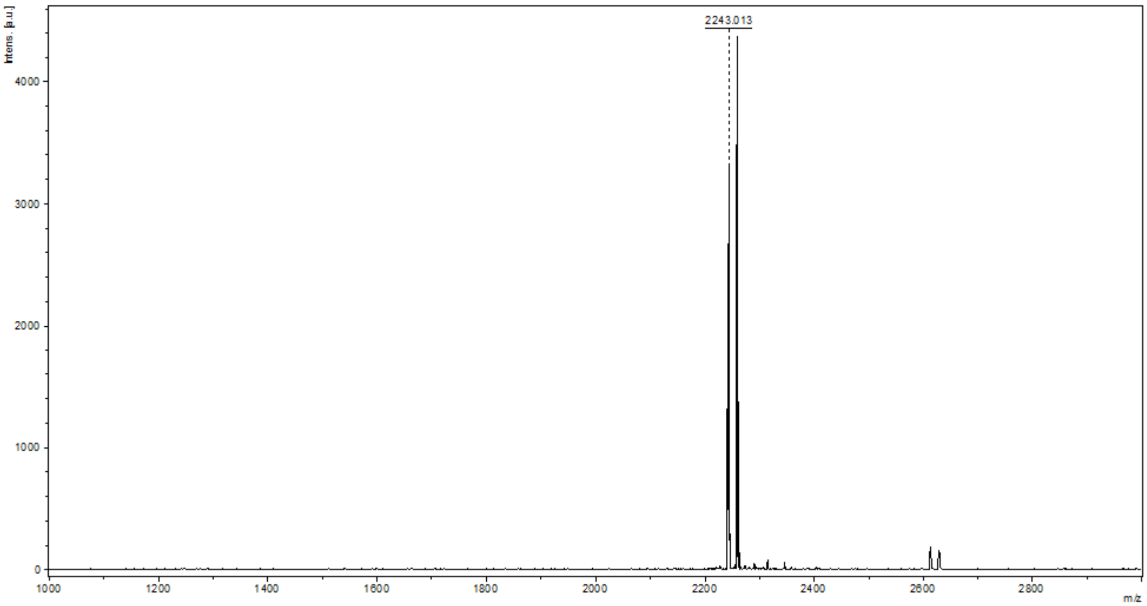

Figure S22 Chemical Structure and MALDI-TOF for Referenced COVID-19 Affinity Ligands

S8 Comparison Table

Table S8. Comparison of Lowest Detectable Spike Protein Concentration by Various Assay System		
**Solid Substrate**	**Capturing Ligand**	**Lowest Detectable Concentration (ng/mL)**
AuNPs	Monoclonal Antibody	48
Au@Pt NPs	Polyclonal Antibody	11
96-well Plates	Monoclonal Antibody	19
Cellulose Nanobeads	Monoclonal Antibody	5
Nanofibrous Membrane	Peptides	1

S9 Reference

[1] Karakuş, E., Erdemir, E., Demirbilek, N., & Liv, L. (2021). Colorimetric and electrochemical detection of SARS-CoV-2 spike antigen with a gold nanoparticle-based biosensor. Analytica chimica acta, 1182, 338939

[2] Fu, Z., Zeng, W., Cai, S., Li, H., Ding, J., Wang, C., ... & Yang, R. (2021). Porous Au@ Pt nanoparticles with superior peroxidase-like activity for colorimetric detection of spike protein of SARS-CoV-2. Journal of Colloid and Interface Science, 604, 113-121.

[3] H. H. Sunwoo, A. Palaniyappan, A. Ganguly, P. K. Bhatnagar, D. Das, A. O. S. El-Kadi, M. R. Suresh, Quantitative and sensitive detection of the SARS-CoV spike protein using bispecific monoclonal antibody-based enzyme-linked immunoassay, J. Virol. Methods. 187 (1) (2013) 72-78.

[4] J.-H. Lee, M. Choi, Y. Jung, S. K. Lee, C.-S. Lee, J. Kim, J. Kim, N. H. Kim, B.-T. Kim, H. G. Kim, A novel rapid detection for SARS-CoV-2 spike 1 antigens using human angiotensin converting enzyme 2 (ACE2), Biosens. Bioelectron. 171 (2021) 112715.
